# Acceptability and Effectiveness of a Fully Web-Based Nutrition and Exercise Program for Individuals With Chronic Disease During COVID-19: Randomized Controlled Trial

**DOI:** 10.2196/57537

**Published:** 2025-03-24

**Authors:** Puneeta Tandon, Kathleen P Ismond, Graeme Purdy, Christofer Cruz, Evelyn Etruw, Kirsten Suderman, Ashley Hyde, Michael Stickland, John C Spence, Dale C Lien, Rahima Bhanji, Carla M Prado, Antonio Miguel-Cruz, Anil A Joy, Maryna Yaskina, Margaret L McNeely

**Affiliations:** 1 Department of Medicine Faculty of Medicine & Dentistry University of Alberta Edmonton, AB Canada; 2 Faculty of Rehabilitation Medicine University of Alberta Edmonton, AB Canada; 3 Faculty of Kinesiology, Sport, and Recreation University of Alberta Edmonton, AB Canada; 4 Department of Agricultural, Food and Nutritional Science Faculty of Agricultural, Life and Environmental Sciences University of Alberta Edmonton, AB Canada; 5 Department of Occupational Therapy Faculty of Rehabilitation Medicine University of Alberta Edmonton, AB Canada; 6 Department of Oncology Faculty of Medicine & Dentistry University of Alberta Edmonton, AB Canada; 7 Women and Childrens' Health Research Institute University of Alberta Edmonton Canada

**Keywords:** eHealth, patient-centered care, adults, geriatrics, self-management, web-based, nutrition, exercise rehabilitation, wearable, activity tracker, quality of life, physical health, 2-minute step test, patients with cancer, chronic diseases, COVID-19, randomized controlled trial, acceptability, effectiveness, intervention

## Abstract

**Background:**

In-person nutrition and exercise interventions improve physical function in chronic diseases, yet the acceptability and effectiveness of web-based delivery, especially with different levels of personnel support, require further investigation.

**Objective:**

This study aims to evaluate a web-based nutrition and exercise intervention delivered entirely digitally from recruitment to trial completion.

**Methods:**

A randomized controlled trial was conducted using the Heal-Me version 1 platform across 2 levels of personnel support (Light and Intensive). Eligible adults with a history of cancer, chronic lung disease, or liver or lung transplant; internet access; and prior participation in a rehabilitation program were enrolled in a fully web-based program to minimize barriers to exercise participation. Participants were randomly assigned (1:1:1) to 1 of 3 study groups. The control group received a detailed, self-directed digital nutrition and exercise guide. The Heal-Me Light group received the web-based intervention alongside dietitian and exercise specialist–led group classes. The Heal-Me Intensive group received web-based intervention, group classes, and one-to-one sessions with the dietitians and exercise specialists. All participants received a wearable activity tracker. The primary acceptability outcome was adherence to the intervention based on a priori targets. The primary effectiveness outcome was the change in Lower Extremity Functional Scale (LEFS) score. Secondary outcomes included physical function tests, which were performed and measured by videoconference. Questionnaires were used to assess well-being, quality of life, and food intake. Analyses adhered to the intention-to-treat principle.

**Results:**

Of 216 participants, 202 (93.5%) completed the intervention (mean 61, SD 11 years; female: 130/202, 64.4%; cancer: 126/202, 62.4%). Adherence exceeded a priori targets, with 82% (105/128) attending >75% of the program elements including postintervention tests. Participants rated the program as “quite a bit” or “very” useful, with similar ratings between Heal-Me Light (56/64, 88%) and Heal-Me Intensive (51/58, 88%) groups (*P*=.69). No significant differences were found for changes in LEFS scores (control: mean 0.8, SD 7.7; Heal-Me: mean 0.3, SD 6.6; *P*=.53). Significant benefits were found in favor of the combined Heal-Me intervention groups versus controls for change in the 2-minute step test, World Health Organization-5 Well-Being Index, Short-Form-36 general, physical health role, energy or fatigue scales, and protein intake. While the change in physical function was similar between the 2 intervention arms, the more intensive one-to-one interaction (Heal-Me Intensive) led to greater improvements in perceived nutrition self-management. No serious adverse events occurred.

**Conclusions:**

The demonstrated satisfaction, adherence, and effectiveness highlight the high acceptability of a web-based, semisupervised nutrition and exercise intervention delivered entirely digitally in individuals with chronic disease. Future studies may benefit from having a baseline physical function inclusion threshold, the use of a more sensitive primary physical function measure, and a higher intensity digital exercise intervention in exercise-experienced participants.

**Trial Registration:**

Clinicaltrials.gov NCT04666558; https://clinicaltrials.gov/study/NCT04666558

**International Registered Report Identifier (IRRID):**

RR2-10.1016/j.cct.2022.106791

## Introduction

Impairments in physical function and physical performance are common in individuals living with serious chronic conditions. Although these impairments increase the risk for morbidity and mortality, they are potentially modifiable with nutrition and exercise rehabilitation [1]. During the COVID-19 pandemic, rehabilitation interventions that were traditionally delivered in person were rapidly transitioned to web-based delivery due to lockdowns and social distancing policies. At the pandemic’s onset, limited data were available to guide the feasibility, safety, and effectiveness of delivering digital nutrition and exercise therapy, and standardized protocols for conducting web-based evaluation of physical function were lacking. Internet-connected digital platforms, such as web-based apps, while offering promise for the scalable delivery of multimodal supportive care interventions, have been associated with poor adherence and retention [2]. Moreover, little information was available to guide how much personnel support was needed to optimize app usage in older patient populations living with chronic disease.

In 2019, our research team co-designed a web-based app (“Heal-Me”) with people living with chronic debilitating diseases, including cancer, cirrhosis, and lung disease [3]. Version 1 of the app was used in this study and is described in more detail in the protocol paper, which includes illustrations of user interface and app features [4]. The Heal-Me web-based app facilitates semisupervised nutrition and exercise programming tailorable to the unique needs of each patient user. It includes nutrition content—a daily protein tracking system, high protein recipes, and cooking videos; exercise content—customizable follow-along exercise videos and autotracking of videos watched; and a calendar for scheduling and accessing web-based group classes and one-to-one check-ins. To guide behavior change, the capability, opportunity, and motivation behavior (COM-B) model and the theoretical domains framework were used as part of the development of the Heal-Me intervention to provide progress tracking, demonstration videos, and graded tasks [4].

With the availability of Heal-Me, this randomized controlled trial (RCT) was designed in response to COVID-19 to answer questions about the acceptability and effectiveness of a personnel-supported, web-based nutrition and exercise intervention for individuals with chronic disease. To understand the extent of support needed by patient users, there were 2 intervention arms (digital group classes only vs digital group classes with additional one-to-one support). We hypothesized that increased contact with a dietitian and exercise professional would result in greater acceptability as measured by higher adherence to the program and greater effectiveness as evaluated by the change in score on the Lower Extremity Function Scale (LEFS).

## Methods

### Study Design

Personalized Online Nutrition and Exercise Routines (PIONEER) was a parallel-group, 3-armed RCT conducted using the Heal-Me web-based program across 12 weeks [4]. The study ran from December 2020 to December 2021, during the COVID-19 pandemic, when Albertan public health policies restricted movement and social interactions. Thus, all trial stages, from recruitment through to end-of-study testing, were performed in a completely digital environment, including participant technology training and the collection of data on physical function.

### Ethical Considerations

The study was approved by the University of Alberta’s Health Research Ethics Board (Pro00103715) and was registered with ClinicalTrials.gov (NCT04666558). Participants provide informed written consent. The privacy and security of the web-based intervention were described previously [4]. There was no monetary compensation provided.

### Recruitment

Study advertisement and recruitment were carried out by the digital distribution of recruitment materials by frontline staff involved in oncology (Alberta Cancer Exercise), pulmonary (Breathe Easy), and posttransplant lung or liver rehabilitation programs (University of Alberta Hospitals) in Edmonton, Alberta. Interested participants were required to contact the research team for further information and eligibility screening. Participants were recruited and enrolled in cohorts that started monthly to facilitate group intervention sessions.

### Participants

Inclusion criteria included adults (aged 18 years and older) with an internet-connected device in their home and the ability to read, write, and speak English. Individuals were eligible if they had cancer (completed an initial course of chemotherapy or radiotherapy but could be on maintenance therapy); had undergone a lung or liver transplant; or had chronic lung disease. All had to graduate from an exercise rehabilitation program before the COVID-19 public health restrictions. The requirement of being an exercise graduate reflected concerns over the safety and complexity of participants having to learn exercise in an entirely web-based environment on top of new tasks (eg, wearable device, learning to use the Heal-Me app, nutrition tracking, and participating in home-based digital physical function assessments) all during the time of COVID-19. Individuals were ineligible if they were receiving compassionate care with an anticipated survival of less than 6 months, had unstable disease status, or were unable to provide informed electronic consent.

### Randomization and Masking

Allocation tables were generated, validated, and uploaded to the REDCap (Research Electronic Data Capture; Vanderbilt University) information system [5] by an independent statistician. Stratification was based on chronic disease status: cancer, lung, or transplant. Randomization was in a 1:1:1 ratio:

- Control (self-directed, digital nutrition and exercise guide)
- Heal-Me Light (Heal-Me app and digital group sessions led by Heal-Me trainers—trainers defined as a registered dietitian and certified exercise specialists including a physiotherapist)
- Heal-Me Intensive (Heal-Me app, digital group sessions, and one-to-one meetings with trainers)

Allocation was concealed from all study staff. The end-of-trial independent assessor was blinded to the participants’ group allocation.

### Procedures

#### Prerandomization Procedures

Web-based activities included advertisement; recruitment; obtaining consent; patient screening; and data collection, including demographics, socioeconomic status, health-related information (eg, medications and comorbidities), and other questionnaires. Outlined in more detail in the protocol [4], the questionnaires included LEFS [6], a validated 20-question survey to assess functional changes in the hip, thigh, knee, leg, ankle, and foot; Physical Activity Readiness Questionnaire [7]; technology proficiency with an internet-connected device (either Computer Proficiency Questionnaire [8] or Mobile Device Proficiency Questionnaire [9]); Unified Theory of Acceptance and Use of Technology [10]; and the Upper Extremity Functional Index-15 [11]. Additional questionnaires were the World Health Organization-5 (WHO-5) Well-Being Index, Generalized Anxiety Disorder-7 [12], De Jong Gierveld Loneliness Scale [13], Short-Form Survey 36-items [14], and self-reported physical activity. The 3-day food records (1 weekend day and 2 weekdays) were completed and emailed to the study coordinator, verified for accuracy by a registered dietitian or dietetic intern, and nutritional intake was analyzed using the FoodProcessor software (version 11.7 .1; ESHA Research). Behavioral beliefs were assessed using the 6-item 0-10 Likert scale for the COM-B [3]. Web-based physical function testing, led by a certified exercise specialist, included the 30-second and 60-second chair sit-to-stands; 1-legged balance; 2-minute step test; shoulder flexion range of motion for left and right sides; and optional tests (if the participants were able and willing), including the chair sit-and-reach and plank test. Height and weight measures were recorded based on patient self-report.

#### Postrandomization Procedures

Participants were randomly assigned and allocated to 1 of 3 study arms after completing the baseline measures. All participants received a wearable activity tracker (Garmin Ltd) to monitor daily step counts, moderate- to vigorous-intensity physical activity minutes, and digital assistance with setup. The self-directed control group received a 52-page manual containing illustrated exercise routines at 3 levels and nutrition guidance (Alberta Health Services and Canada’s Food Guide).

Participants in the Heal-Me Light and Heal-Me Intensive interventions had three web-based sessions as detailed in the protocol [4]: (1) an introduction to and instruction in the use of the Heal-Me app; (2) a dietitian-led nutrition assessment, review of nutrition preferences, and the setting of a protein goal target in the app (1.2-1.5 g/kg/day based on guidelines [15-17]); and (3) a certified exercise specialist–led review of home space for exercise, review of exercise preferences, and setting up of an individualized program in the app. While the focus of nutrition counseling was on protein intake, a condition-specific, guideline-based target for daily caloric intake of 25-30 kcal/kg/day was also presented in the app, and counseling was provided about eating a balanced plate [15-17]. Recognizing the limited data supporting the safety of digital exercise, the certified exercise specialist started all participants at a low-intensity level for exercise. Participants were asked to complete 45-60 minutes of exercise sessions 3 times a week (2 independent sessions and 1 certified exercise specialist–led, web-based group class), track protein intake for 3 days each week, and attend 5 web-based group nutrition classes over the 12-week study. Heal-Me Intensive participants also received one-to-one check-ins with the dietitian (2×, 20-30 minutes) and certified exercise specialist (5×, 20-30 minutes) over the 12-week study period.

After completing the 12-week trial, participants repeated web-based physical function assessments, anthropomorphic measures, questionnaires, and 3-day food records and completed the study satisfaction survey.

### Study Objectives

#### Acceptability

This was comprised of adherence rate and satisfaction. Our primary acceptability outcome was adherence, which was defined as completing more than 75% of the group nutrition and exercise sessions and completing all end-of-study outcomes. Satisfaction was collected from the end of the study program experience and satisfaction questionnaire. Two questions inquired about whether intervention participants felt prepared to continue their nutrition and exercise activities after program completion. Using a 5-item Likert scale (from 1=not at all to 5=very much), participants were asked “Do you feel that the PIONEER nutrition program has prepared you to reach your target protein goal on your own?” and “Do you feel that the PIONEER exercise program has prepared you to reach your exercise goal on your own?” Technology acceptability was evaluated with the Unified Theory of Acceptance and Use of Technology and will be presented in a follow-on publication.

#### Effectiveness

This was comprised of a battery of objective outcomes including physical function tests and physical activity, and self-reported outcomes including quality of life. Our primary effectiveness outcome was the change in LEFS score from baseline to 12 weeks between the control and combined intervention arms. Secondary objective effectiveness outcomes included the 12-week change in objective physical function comparing the control and intervention groups for the 2-minute step test, chair sit-to-stand (30 and 60 seconds), plank, shoulder range of movement, and 1-legged balance. Step count and moderate- to vigorous-intensity physical activity minutes for weeks 2 to 11 were averaged for each week. Data from weeks 1 and 12 were not considered because postal delivery and setup of the device often extended into the first week, and device return occurred at the end of week 12. Secondary self-reported effectiveness outcomes included Upper Extremity Functional Index-15, mental health, and quality of life (ie, WHO-5 Well-Being Index, Generalized Anxiety Disorder-7, De Jong Gierveld Loneliness Scale, and Short-Form-36). Adverse events were recorded by study staff.

#### Exploratory Outcomes (Baseline and 12 Weeks)

Changes in daily calorie, protein intake, and the proportion of participants reaching target protein intake (>1.2 g/kg/day protein based on ideal body weight using a BMI of 24.9 kg/m^2^) were evaluated. The COM-B scale assessed changes in behavioral beliefs, with data planned for presentation in a future publication. For physical activity, the validated Single Item Physical Activity question was used, where participants were asked “In the past week, on how many days have you done a total of 30 minutes or more of physical activity, which was enough to raise your breathing rate?” [18]. Additionally, we explored results comparing the highest and lowest quartiles of baseline function for the 2-minute step test.

### Sample Size

The sample size and statistical analysis plans are presented in detail in the protocol [4]. The primary analysis used the ANOVA model to assess the difference in the LEFS scores between the 3 study groups. To preserve type 1 error in 3 between-group comparisons, the α was adjusted by the Bonferroni correction. Thus, with a 2-sided α=.05 (.0167 adjusted for multiple comparisons) and β=.20 (80% power), 60 participants per group (180 in total) were estimated to be required to determine a statistically significant difference for a moderate (0.6) Cohen *d* effect size (minimal clinically important difference of 9 points [19]). To account for a conservative 20% dropout rate, the total sample size was increased to 216 (n=72 per arm).

### Statistical Analysis Plan

The intention-to-treat principle was used for all analyses. Descriptive statistics were used to understand the participants’ characteristics and outcome variables. The between-groups comparison was analyzed using ANOVA (or its nonparametric alternative) and the Tukey honest significant difference test for pairwise comparisons using a familywise α of .05. The primary analysis, the LEFS score, was then analyzed by linear mixed models with random effects, which were adjusted for the baseline score (T1) as a covariate [20,21]. A 3-group comparison was planned only if the combined 2-group comparison was significant. Exercise adherence rates (proportion completing 80% of exercise sessions over the study) and dietary protein adherence rates (proportion meeting 80% of target intake over the study) were compared between study groups by the chi-square test. The differences in the frequency of adverse events were assessed using the chi-square test. Linear regressions were constructed to determine clinically significant outcomes, adjusted for sex, age, and comorbidity status; group assignment was an independent variable. Missing data resulted in listwise deletion of the cases. Analyses were performed using either SAS (version 9.4 or later; SAS Institute) or SPSS (version 28 or later; IBM Corp).

## Results

### Participant Characteristics

**Figure 1:**
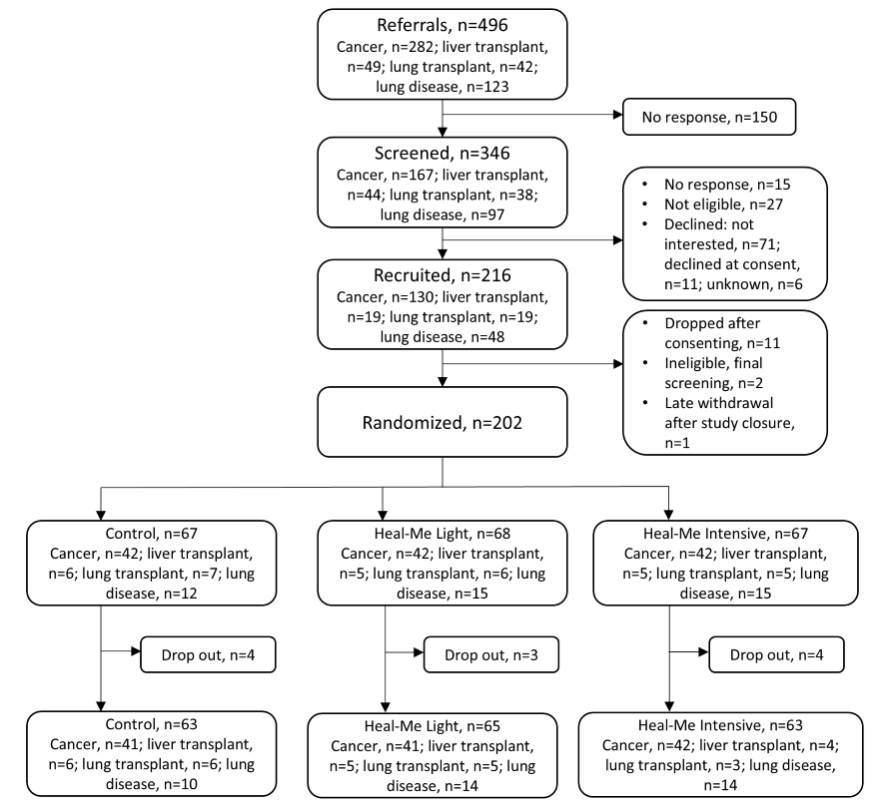
CONSORT (Consolidated Standards of Reporting Trials) patient flowchart.

The 202 participants (Figure 1) were recruited over 10 months, and none were screened out for unstable disease. They had a mean age of 61.0 (SD 11.1) years and 64.4% (130/202) were female. At baseline, 83.2% (168/202) attended postsecondary education, and 44.5% (90/202) earned more than CAD $80,000 (US $ conversion rate for 2023: US $1=CAD $1.3497) per annum (Table 1). The most common chronic condition was cancer (126/202, 62.4%), while the least common was liver transplantation (16/202, 7.9%). The level of comorbidity was high, with a mean Charlson Comorbidity Index of 4.6 (SD 2.0), and the mean BMI was 28.4 (SD 6.2) kg/m^2^. The mean technology proficiency measured with the Computer Proficiency Questionnaire and Mobile Device Proficiency Questionnaire was 88.7% (SD 14.9%).

**Table 1:** Participant characteristics at baseline.

Characteristic		Overall (n=202)	Control (n=67)	Heal-Me Light (n=68)	Heal-Me Intensive (n=67)
Age (years), mean (SD)		61.0 (11.1)	61.2 (9.4)	61.4 (11.7)	60.4 (12.1)
**Sex, n (%)**					
	Female	130 (64.4)	44 (65.7)	47 (69.1)	39 (58.2)
	Male	72 (35.6)	23 (34.3)	21 (30.9)	28 (41.8)
**Education, n (%)**					
	Some or completed high school	34 (16.8)	11 (16.4)	14 (20.6)	9 (13.4)
	Some university or college	33 (16.3)	6 (8.9)	14 (20.6)	13 (19.4)
	Completed university or college	95 (47.0)	34 (50.7)	29 (42.6)	32 (47.8)
	Some or completed graduate school	40 (19.8)	16 (23.9)	11 (16.2)	11 (16.2)
**Annual income (CAD $)^a^, n (%)**					
	Less than 20,000	9 (4.4)	1 (1.5)	6 (8.8)	2 (3)
	Between 20,000 and 39,000	30 (14.8)	11 (16.4)	10 (14.7)	9 (13.4)
	Between 40,000 and 59,000	28 (13.9)	14 (20.9)	8 (11.8)	6 (9)
	Between 60,000 and 79,999	26 (12.9)	9 (13.4)	7 (10.3)	10 (14.9)
	Between 80,000 and 99,999	31 (15.3)	9 (13.4)	13 (19.1)	9 (13.4)
	More than 100,000	59 (29.2)	21 (31.3)	19 (27.9)	19 (28.3)
	Not reported	19 (9.4)	2 (3)	5 (7.3)	12 (17.9)
**Ethnicity, n (%)**					
	Arab	1 (0.5)	1 (1.5)	0 (0)	0 (0)
	Asian	12 (5.9)	4 (6)	3 (4.4)	5 (7.5)
	Indigenous or First Nations	3 (1.5)	1 (1.5)	2 (2.9)	0 (0)
	Latin or Central or South American	4 (2)	1 (1.5)	1 (1.5)	2 (3)
	White	174 (86.1)	57 (85.1)	60 (88.2)	57 (85.1)
	Not reported	8 (4)	3 (4.5)	2 (2.9)	3 (4.5)
**Marital status, n (%)**					
	Never married	24 (11.9)	8 (4.0)	9 (4.5)	7 (3.5)
	Married or common law	136 (67.3)	47 (40.3)	41 (60.3)	48 (71.6)
	Divorced or separated	28 (13.9)	8 (4)	15 (22)	5 (2.5)
	Widowed	14 (6.9)	4 (2)	3 (1.5)	7 (3.5)
**Disease group, n (%)**					
	Cancer	126 (62.4)	42 (62.7)	42 (61.8)	42 (62.7)
	Lung disease	42 (20.8)	12 (17.9)	15 (22.1)	15 (22.4)
	Lung transplant recipient	18 (8.9)	7 (10.4)	6 (8.8)	5 (7.5)
	Liver transplant recipient	16 (7.9)	6 (8.9)	5 (7.3)	5 (7.5)
Charlson Comorbidity Index, mean (SD)		4.6 (2)	4.8 (2.1)	4.5 (1.9)	4.5 (2.2)
Current smokers, n (%)		5 (2.5)	3 (4.5)	1 (1.5)	1 (1.5)
BMI baseline (kg/m^2^; n=198), mean (SD)		28.4 (6.2)	28.7 (6.8)	28.1 (5.5)	28.4 (6.2)
Hemoglobin (g/L; n=191), mean (SD)		133.7 (16.7)	132.6 (18.9)	133.2 (15)	135.4 (16)
Physical Activity (minutes/week) Single question self-report (n=200), mean (SD)		101.7 (86)	105.5 (66.6)	85.6 (62)	114.5 (117.4)

^a^US $ conversion rate for 2023: US $1= CAD $1.3497.

### Study Objectives

#### Acceptability

The completion rate for the study was 94.6% (191/202), and the mean attendance for arms 2 (n=66) and 3 (n=62) combined was 78.6% for the 5 group nutrition classes, 88.5% for the 12 group exercise sessions, and 78.8% for the home exercise videos, with no statistically significant differences (*P*=.84, *P*=.64, and *P*=.67, respectively) between the 2 intervention arms. In response to the statement “Completing the PIONEER program helped me to meet my health and wellness goals,” 82.8% (101/122) of participants in Heal-Me Light and 90.2% (110/122) of participants in Heal-Me Intensive answered “quite a bit” to “very much” (2 of the 5 highest categories), with no significant difference between arms (*U*=1920; *z*=0.41; *P*=.69). Although staff services were scored as “excellent” (97/122, 79.5%), participants in Heal-Me Intensive scored staff services statistically significantly higher than participants in Heal-Me Light (*U*=2209; *z*=2.57; *P*=.01). At the end of the study, when participants were asked if PIONEER had prepared them to reach their exercise and protein goals after program completion; for exercise, 81.1% (99/122) responded either “quite a bit” or “very much,” and for nutrition, 82.6% (100/121) responded either “quite a bit” or “very much.” Those in Heal-Me Light (mean rank 53.6) reported significantly less preparedness to reach their daily protein intake than those in Heal-Me Intensive (mean rank 69.3; *U*=2298; *z*=2.66; *P*=.008). No significant difference between interventional groups was found regarding achieving exercise goals (59.6 vs 63.7; *P*=.495). Overall, participants reported that their program was “quite a bit” or “very” useful (top 2 out of 5 categories) for Heal-Me Light (56/64, 88%) and Heal-Me Intensive (51/58, 88%; *U*=1920; *z*=0.41; *P*=.69). Most participants would recommend the program either “quite a bit” or “very much” (top 2 out of 5 categories) to others living with a chronic disease (Heal-Me Light: 59/64, 92% and Heal-Me Intensive: 57/58, 98.3%; *U*=1843; *z*=–1.00; *P*=.92). More generally, when asked about the perceived burden of doing the web-based physical function assessments, 73.8% (90/122) reported “no burden at all.”

#### Effectiveness

##### Primary Effectiveness Outcome: LEFS

The baseline LEFS scores for the control and Heal-Me groups were 63.6 (SD 13.9) and 62.9 (SD 14.5), respectively. The difference of –0.53 (95% CI –2.7 to 1.7) was not statistically significant between groups for self-reported physical function, even when analyzed by the disease group (Table 2 and Table S1 in Multimedia Appendix 1).

**Table 2:** Primary and secondary fitness outcomes.

Measure		T1 (baseline), mean (SD)		T2 (end-of-study), mean (SD)		Change from T2 to T1, mean (SD)	Unadjusted between-group differences: T2 to T1, mean (95% CI)			Adjusted^a^ between-group differences: T2 to T1, mean (95% CI)		
**LEFS^b^ score, (n=177)**									0.53 (–1.7 to 2.7)			0.60 (–1.6 to 2.8)
	Control		63.6 (13.9)		64.4 (14.3)	0.8 (7.7)						
	Heal-Me app		62.9 (14.5)		63.5 (14.5)	0.3 (6.6)						
**2-minute step test (n=171)**									10.0 (4.6 to 15.4)^c^			9.8 (4.7 to 15.0)^c^
	Control		80.5 (23.9)		81.9 (23.1)	1.3 (15.5)						
	Heal-Me app		79.6 (21.0)		91.0 (22.3)	11.3 (17.4)						
**Chair sit-to-stand 60 seconds (n=171)**									0.43 (–1.1 to 1.9)			0.22 (–1.3 to 1.7)
	Control		29.7 (11.3)		30.3 (10.9)	0.6 (4.0)						
	Heal Me app		27.5 (9.6)		28.5 (9.8)	1.0 (4.9)						
**Chair sit-to-stand 30 seconds ( n=171)**									0.16 (–0.67 to 0.98)			0.02 (–0.78 to 0.83)
	Control		15.6 (5.8)		15.8 (5.5)	0.16 (2.1)						
	Heal-Me app		14.6 (4.9)		15.0 (4.8)	0.32 (2.7)						
**Sit-and-reach (cm reached, n=61)**									–1.53 (–4.7 to 1.6)			–1.6 (–4.8 to 1.6)
	Control		–4.1 (12.9)		–0.9 (13.2)	3.2 (4.3)						
	Heal-Me app		–3.6 (12.6)		–2.0 (13.1)	1.7 (6.2)						
**Shoulder range of motion right (degrees, n=160)**									–1.4 (–4.0 to 1.2)			–0.5 (–2.9 to 1.9)
	Control		150 (8.1)		152 (10.3)	2.0 (8.5)						
	Heal-Me app		153 (12.4)		153 (11.1)	0.6 (7.5)						
**Shoulder range of motion left (degrees, n=160)**									–0.05 (–2.5 to 2.5)			0.4 (–1.9 to 2.8)
	Control		149 (11.0)		150 (12.4)	0.87 (6.5)						
	Heal-Me app		151 (12.6)		152 (11.5)	0.82 (8.0)						
**Balance right (seconds, n=172)**									2.7 (–0.6 to 6.1)			3.0 (–0.6 to 6.1)
	Control		29.2 (18.0)		29.2 (17.5)	0.0 (9.8)						
	Heal-Me app		29.8 (15.7)		32.6 (15.2)	2.8 (10.6)						
**Balance left (seconds, n=172)**									1.2 (–2.4 to 4.6)			1.5 (–1.7 to 4.8)
	Control		29.6 (16.4)		30.7 (16.2)	1.1 (11.3)						
	Heal-Me app		31.2 (15.6)		33.4 (15.0)	2.2 (10.6)						
**Plank (seconds, n=134)**									8.1 (–3.5 to 19.6)			7.7 (–4.0 to 19.4)
	Control		63.4 (42.4)		75.3 (49.9)	11.9 (22.5)						
	Heal-Me app		67.2 (56.3)		87.2 (69.7)	20.0 (35.1)						

^a^Adjusted for age, Charlson Comorbidity score, and baseline value.

^b^LEFS: Lower Extremity Functional Scale.

^c^*P*<.001 between-group improvement from T2 to T1.

##### Secondary Objective Outcomes

A statistically significant, between-group difference was found in favor of the intervention for the 2-minute step test (*P*<.001). Details on the web-based assessed changes in physical function outcomes are presented in Table 2 and Table S1 in Multimedia Appendix 1. The intervention and control groups had similar step counts and moderate- to vigorous-intensity physical activity minutes as measured by the wearable activity tracker (Figures S1 and S2 in Multimedia Appendix 1).

#### Secondary Self-Reported Outcomes

Changes in the WHO-5 Well-Being Scale (*P*=.02) and the Short-Form-36 general health (*P*=.04) were statistically significantly different between the control and combined intervention arms according to 2-tailed independent *t* tests (Table 3; Table S2 in Multimedia Appendix 1).

**Table 3:** Participant-reported outcomes between control and Heal-Me app (Light and Intensive).

Measure		T1 (baseline), mean (SD)	T2 (end-of-study), mean (SD)	Change from T2 to T1, mean (SD)	Unadjusted between-group differences: T2 to T1, mean (95% CI)		Adjusted^a^ between-group differences: T2 to T1, mean (95% CI)	
**Upper Extremity Functional Index (n=187)**						1.72 (0.01-3.4)^b^		1.6 (–0.07 to 3.2)
	Control	72.1 (11.3)	71.1 (11.0)	–0.95 (5.7)				
	Heal-Me app	71.7 (9.5)	72.4 (9.7)	0.80 (5.5)				
**WHO-5^c^ Well-Being Scale (n=190)**						5.5 (1.3-9.6)^d^		4.94 (0.8-9.1)^e^
	Control	63.5 (16.7)	62.6 (18.2)	–0.97 (12.3)				
	Heal-Me app	62.0 (21.8)	66.5 (20.9)	4.53 (15.5)				
**SF-36^f^ general (n=190)**						3.83 (0.13-7.53)^g^		3.74 (0.13-7.35)^g^
	Control	58.5 (17.4)	58.3 (19.7)	–0.24 (12.3)				
	Heal-Me app	58.1 (20.4)	61.6 (20.5)	3.59 (12.1)				
**SF-36 physical functioning**						0.09 (–3.9 to 4.11)		0.27 (–3.6 to 4.2)
	Control	72.4 (23.4)	73.4 (22.0)	0.97 (13.4)				
	Heal-Me app	70.6 (22.9)	71.7 (23.8)	1.05 (13.1)				
**SF-36 physical health role**						16.9 (4.6 to 20.3)^h^		16.3 (5.3 to 27.4)^i^
	Control	56.0 (42.1)	49.6 (44.9)	–6.4 (43.1)				
	Heal-Me app	54.9 (40.2)	65.4 (40.8)	10.5 (39.2)				
**SF-36 emotional health role**						10.4 (–1.6 to 22.3)		9.7 (–0.04 to 13.4)
	Control	65.6 (38.1)	67.2 (37.9)	1.6 (38.8)				
	Heal-Me app	65.1 (36.7)	77.1 (33.4)	11.9 (39.6)				
**SF-36 energy or fatigue**						4.63 (0.29 to 9.0)^j^		4.81 (0.72 to 8.90)^k^
	Control	55.3 (21.1)	54.7 (19.9)	–0.64 (13.8)				
	Heal-Me app	56.9 (20.8)	60.9 (22.0)	3.98 (14.4)				
**SF-36 emotional well-being**						3.32 (–0.34 to 7.0)		2.44 (–0.86 to 5.7)
	Control	77.1 (14.5)	76.8 (15.2)	–0.26 (11.6)				
	Heal-Me app	74.9 (158)	78.0 (14.4)	3.06 (12.2)				
**SF-36 social functioning**						5.0 (–1.0 to 11.0)		2.5 (–2.3 to 7.3)
	Control	80.2 (21.2)	82.5 (18.5)	2.2 (19.3)				
	Heal-Me app	75.6 (23.9)	82.8 (20.1)	7.2 (19.9)				
**SF-36 pain**						0.73 (–4.9 to 6.3)		0.02 (–5.1 to 5.1)
	Control	73.5 (22.1)	72.0 (21.6)	–1.5 (17.3)				
	Heal-Me app	71.8 (19.9)	71.0 (20.4)	–0.8 (18.9)				
**GAD-7^l^ (anxiety, n=190)**						–0.31 (–1.46 to 0.85)		–0.64 (–1.4 to 0.12)
	Control	3.4 (3.5)	3.6 (3.6)	0.16 (2.8)				
	Heal-Me app	3.8 (3.9)	3.2 (3.9)	–0.56 (2.6)				
**Loneliness Scale (n=190)**						–0.07 (–0.61 to 0.47)		–0.05 (–0.4 to 0.3)
	Control	2.2 (1.8)	2.4 (1.8)	0.18 (1.2)				
	Heal-Me app	2.3 (1.8)	2.3 (1.7)	0.09 (1.4)				

^a^Adjusted for age, Charlson comorbidity score, and baseline value.

^b^*P=*.049.

^c^WHO-5: World Health Organization-5.

^d^*P=*.009.

^e^*P=*.02.

^f^SF-36: Short-Form-36.

^g^*P=*.04.

^h^*P=*.007.

^i^*P=*.004.

^j^*P=*.04.

^k^*P=*.02.

^l^GAD-7: Generalized Anxiety Disorder-7.

#### Adverse Events

No serious adverse events occurred during the trial. However, 33 minor health or musculoskeletal complaints (eg, muscle pain) were reported among participants in the 3 study groups (control: 10/33, 30.3%; Heal-Me Light: 11/33, 30.3; Heal-Me Intensive: 12/33, 39.4%; *P*=.71). A total of 21 (63.6%) adverse events were reported as “clearly unrelated,” 5 (15.1%) as “possibly related,” and only 5 (15.1%) as “related” to study participation.

#### Exploratory Outcomes

A total of 144 (71.2%) participants had a 3-day food record completed at both baseline and the end of study. Analysis of the food records showed that protein and calorie intakes increased in the Heal-Me intervention arms (Heal-Me Light and Intensive) compared to controls (Table S3 in Multimedia Appendix 1). After adjusting for age and comorbidity, the between-group improvements from baseline were presented as mean differences (95% CI) for calories, 246 kcal/kg/d (95% CI 57-436), and protein, 22.8 g/kg/d (95% CI 12.5-33.1). The proportion of participants at goal protein intake (1.2 g/kg/d) at baseline was 53% (23/43) in the control group and 37.6% (38/101) in Heal-Me. By week 12, a total of 53.5% (23/43) of the control group and 81.2% (82/101) of participants in Heal-Me had reached the target protein intake (*P*<.001). Similar proportions of participants in Heal-Me reached the target protein intake (*P*=.53).

In our exploratory analyses of the lowest and highest quartiles of the 2-minute step test, a significant difference was found in favor of Heal-Me Light and Intensive (Figure S3 in Multimedia Appendix 1). For the lowest quartile of participants, the mean difference between the control and intervention groups for the 2-minute step test was 26.3 steps (95% CI 2.24-50.4; *P=*.03) favoring the intervention, as compared to 5.8 steps (95% CI –0.88 to 12.37; *P*=.09) in the highest quartile (Figures S1 and S2 in Multimedia Appendix 1).

## Discussion

### Principal Findings

To the best of our knowledge, this is the largest, 3-armed, semisupervised nutrition and exercise RCT that has been completed entirely on the web from recruitment to end-of-trial assessments. Acceptability was high across both adherence and satisfaction measures, but the primary effectiveness outcome of self-reported lower extremity function was not significantly different between groups. Statistically significant improvements were found in the Heal-Me intervention for the 2-minute step test, well-being, quality of life, and the proportion of participants reaching target protein intake. Notably, the trial was conducted at a unique time point in our history, against a background of COVID-19 community lockdowns and its associated uncertainties, as well as at the beginning of a now increasing number of studies [22] that are leveraging app-based technologies to support nutrition and exercise interventions.

Acceptability, a primary objective of this study, was met with high rates of adherence and satisfaction. The adherence rate of 82% (105/128) exceeded our a priori level of 75% and is particularly noteworthy given the complexity of the intervention in combination with its fully web-based delivery and evaluation. An in-person prehabilitation intervention of similar complexity (exercise, nutrition, smoking cessation, and psychological support), albeit only 4 weeks in duration, reported that 77% of their 251 participants completed at least 75% of their exercise sessions [23]. In contrast, fully digital prehabilitation interventions have met with lower rates of adherence. In a 4-week digital prehabilitation nutrition and exercise app for colorectal cancer, only 56% (127/227) of participants met the adherence metric of being accessed at least once during the study [24]. In another app-based study of liver transplant candidates (n=31; mean 61, SD 7 years), all received weekly telephone support from a remote physical activity coach during a 12-week physical fitness app intervention [25]. Despite the one-to-one engagement with a trainer, 57% (18/31) of the participants were considered adherent. Consistent with these adherence challenges, a review of supervised and unsupervised mobile health interventions reported an overall adherence of only 56% (55/99), 49% (49/99) for nutrition apps, and 55% (54/99) for physical activity apps [2].

The reasons for the high adherence rates in this study are likely multifaceted. The concept of Heal-Me began with a patient focus group followed by regular interactions with patients and providers, usability or user experience testing, and a pilot study in multiple myeloma, all of which helped shape the app’s design, content, and program delivery [3,26]. While there is limited evidence describing the impact of co-design on experimental outcomes, the acceptability and adherence of the Heal-Me app are at least partially attributable to patient and provider involvement across all stages. Moreover, the app included many of the features inventoried in 2 recent systematic reviews for promoting adherence and satisfaction, including ease of use, access to health care information, real-time supervision and monitoring, as well as personalization and automated reminders for calendar events [2,27].

The effectiveness of the intervention was our second objective. The primary outcome of LEFS score was nonsignificant when compared to the control group. Although this may represent a true lack of effectiveness of multimodal intervention, the lack of significance may also be due to the choice of the LEFS as the primary physical function outcome measure. As a web-based tool, it was selected because of its wide use in populations with musculoskeletal conditions [28] and its association with quality of life, the 6-minute walk test, and clinical outcomes including hospital admissions and falls [4]. As such, during COVID-19, it provided a suitable option over more commonly administered, in-person physical function tests. The normative score of the LEFS is 63 for both male and female individuals aged 60-64 years, with a maximum value of 80 and a minimal clinically important difference of 9 points [28]. In this study, the LEFS score at baseline was already at a mean level of 63 (SD 14.1; n=202), potentially creating a ceiling effect that may have impacted the potential for improvement [23]. Objective measures of physical function are seen as more sensitive indicators of functional abilities [29] and were assessed at baseline and the end of study digitally as secondary outcome measures. A significant between-group difference was found for the 2-minute step test. Of note, unlike the LEFS, this test may have been more sensitive to change as baseline values were more compromised, in the range of the 25th-50th percentile of adults aged 60 to 64 years. In our exploratory analysis of the 2-minute step test, a significant between-group difference was found when comparing results from the lowest and highest quartiles, suggesting primary benefit for those with lower physical functioning, and pointing to future rationale for stratifying inclusion by baseline physical function.

Compared to the control group, the combined intervention group showed statistically significant benefits in some of the quality-of-life measures, which remained when adjusting for age, comorbidity, and baseline values. While the overall sample was comprised of individuals motivated to participate in a nutrition and exercise–supported, web-based program, the average step count, physical activity minutes, and protein intake across groups still fell below the levels recommended by current Canadian public health guidelines [15-17,30].

The study included 2 intervention arms for which we hypothesized that given sufficient statistical power, increased contact with a dietitian and exercise professional would demonstrate greater acceptability and greater effectiveness. The group and one-to-one sessions required a weekly time commitment of approximately 80 minutes in Heal-Me Light and 100 minutes in Heal-Me Intensive in addition to the nutrition tracking and independent exercise. Despite lengthy time commitments, there were similarly high rates of satisfaction and adherence in both groups. This suggests that access to the app and group sessions may have been sufficient to drive acceptability. Unfortunately, the limited differences in the effectiveness outcomes between the intervention and control groups made it impossible to assess the impact of the different levels of personnel support on these measures. While regular interactions with staff and progress monitoring have been previously identified as beneficial facilitators in studies, including those for cardiac telerehabilitation [31] and upper gastrointestinal cancer surgery [32], future studies are encouraged to explore the level of personnel interaction that maximizes participant acceptability and effectiveness.

While adequately powered, the short duration of the study in combination with the heterogeneity across multiple chronic diseases of participants may have contributed to the lack of difference in mean values between the study groups. Furthermore, while biases in self-reporting may have resulted due to social desirability between the participant and staff members, the digital nature of the survey tools was used to minimize bias [33].

This RCT adds to accumulating evidence showing that web-based physical function measures are feasible to conduct. While some data are available to suggest minimal differences between in-person and web-based measures, their validity remains uncertain. In 2023, Heslop et al [34] reviewed 17 studies comparing digital to in-person physical performance test results in older adults. In the 9 studies that assessed digital compared to in-person results, accuracy was reported as “good” (≤5% difference) in 6 studies, “moderate” (5%-10% mean difference) in 2 studies, and “poor” (>10% mean difference) in 1 study. Studies by Guidarelli et al [35] (n=176) and Hoenemeyer et al [36] (n=112) found moderate to strong agreement between remotely evaluated tests with in-person testing. While participants in our study reported little burden with the web-based testing (10% found it “somewhat” or “quite a bit” burdensome), additional certified exercise specialist staff were required to support digital exercise classes for safety. For the web-based physical function tests, two research team members were needed: one to manage the technology (eg, camera view, sound) and lead the test, and the other to score the test.

### Limitations

A key limitation of the study was participant selection. Although carried out with sound rationale, we limited enrollment to participants who had graduated from an exercise-based rehabilitation program for their respective chronic diseases. The history of previous exercise participation is associated with self-effectiveness, competence, and adherence to exercise interventions [37,38]. The step count and moderate- to vigorous-intensity physical activity minutes were similar for the intervention and control groups, suggesting a similar degree of physical activity in the control group [39]. Potential contributors that facilitated the control arm exercise were baseline experience with exercise, the provision of an extensive 52-page nutrition and exercise handout, and a wearable activity tracker; the latter are known to increase step count outcomes in adults over the short term [40]. Despite this contamination, improvements were seen in favor of the Heal-Me–supported programming for self-reported health and quality-of-life measures.

Regarding the nutrition component, while significant improvements were seen in target protein intake, verification of the food records was a time-consuming task and frequently involved patient contact for clarification or additional information. Also, challenges were noted with participants submitting their 3-day food records, with only 71.2% with complete records both at baseline and the end of study, suggesting the need to explore additional forms of tracking in future studies [41]. In addition, a completely web-based exercise testing and intervention delivery model was a new experience for our team. Our digital exercise programs, in retrospect, erred on the side of safety regarding intensity and duration, and for this group of experienced participants, may have been too conservative. Given the multiple secondary outcomes, we acknowledge the potential for type 1 errors. Finally, because of the limited differences seen in outcomes between the intervention and control groups, we were unable to assess the impact of the different levels of personnel support, a factor of interest in future studies.

The Heal-Me PIONEER acceptability and effectiveness trial offers many learnings. As a large trial completed fully digitally during a unique period in our history, it demonstrates the feasibility of rapid recruitment, web-based testing and intervention, and high acceptability and adherence rates among a mixed population with chronic conditions. A comprehensive battery of tests was performed, providing reference points for future studies using the same measures. We also offer considerations for modifications based on our learnings, including stratification for physical function; the selection of a more sensitive objective physical function measure; and the provision of a more tailored, higher-intensity, web-based exercise intervention. In this burgeoning age of digital health, characterized by an increasing reliance on digital assessments and rehabilitative interventions, our findings underscore the feasibility of widespread implementation of nutrition and exercise programming, alongside high participant acceptability and adherence.

## Appendix

Multimedia Appendix 1 Supporting materials.

Multimedia Appendix 2 CONSORT-eHealth (V 1.6.1) checklist.

## Abbreviations

COM-B: capability, opportunity, and motivation behavior
LEFS: Lower Extremity Functional Scale
PIONEER: Personalized Online Nutrition and Exercise Routines
RCT: randomized controlled trial
REDCap: Research Electronic Data Capture
WHO-5: World Health Organization-5

## Data Availability

The datasets generated during and/or analyzed during this study are not publicly available due to data sharing policies but are available from the corresponding author on reasonable request.
