# Supplementary material for: Acceptability and Effectiveness of a Fully Web-Based Nutrition and Exercise Program for Individuals With Chronic Disease During COVID-19: Randomized Controlled Trial
Source: J Med Internet Res. 2025 Mar 24;27:e57537. doi: 10.2196/57537 (PMC11976182; doi:10.2196/57537)
Supplement: Multimedia Appendix 1 [file jmir_v27i1e57537_app1.docx]

**Table S1.** Exploration of 3 arms of the statistically significant fitness outcome variable from Table 2.

|  | T1  (Baseline) | T2  (End-of-study) | Change  T2-T1 | Unadjusted between-group differences: T2 to T1 | | |
| --- | --- | --- | --- | --- | --- | --- |
| Measure | Mean (SD) | Mean (SD) | Mean (SD) | Arm 3 vs Arm 1  Mean (95% CI) | Arm 2 vs Arm 1  Mean (95% CI) | Arm 3 vs Arm 2  Mean (95% CI) |
| 2-Minute Step Test  Arm 1 - control  Arm 2 - light  Arm 3 - intensive | 80·5 (23·9)  79·4 (21·8)  79·8 (20·1) | 81·9 (23·1)  89·8 (22·6)  92·1 (22·1) | 1·3 (15·5)  10·4 (16·5)  12·3 (18·4) ^a^ | 11·0 (3·3, 19·7) ^b^ | 9·1 (1·5, 16·7) ^b^ | 1·9 (-5·7, 9·5) |

^a^ *P*<.05 within group improvement T2 to T1

^b^ *P*<.05 between group improvement T2 to T1

**Table S2.** Exploration of 3 arms of the statistically significant outcome variables from Table 3.

|  | T1  (Baseline) | T2  (End-of-study) | Change  T2-T1 | Unadjusted between-group differences: T2 to T1 | | |
| --- | --- | --- | --- | --- | --- | --- |
| Measure | Mean (SD) | Mean (SD) | Mean (SD) | Arm 3 vs Arm 1  Mean (95% CI) | Arm 2 vs Arm 1  Mean (95% CI) | Arm 3 vs Arm 2  Mean (95% CI) |
| SF-36 General Health  Arm 1 - control  Arm 2 - light  Arm 3 - intensive | 58·5 (17·3)  57·0 (20·9)  59·1 (19·9) | 58·3 (19·7)  62·7 (20·4)  60·6 (20·7) | -0·2 (12·3)  5·7 (11·4) ^a^  1·4 (12·4) | 1·7 (-3·5, 6·9) | 5·9 (0·8, 11·1) ^b^ | -4·2 (-9·4, 0·9) |
| WHO-5 Well-Being  Arm 1 - control  Arm 2 - light  Arm 3 - intensive | 63·4 (16·6)  63·0 (23·0)  61·1 (20·5) | 61·6 (19·7)  67·7 (18·9)  64·3 (24·0) | -1·8 (13·8)  4·7 (17·4) ^a^  3·2 (16·1) | 5·0 (-1·8, 11·8) | 6·5 (-0·3, 13·3) | -1·6 (-8·3, 5·2) |

^a^ *P*<.05 within group improvement T2 to T1

^b^ *P*<.05 between group improvement T2 to T1

**Table S3.** Exploratory outcomes between control (arm 1) and Heal-Me app (arms 2 & 3).

|  | T1  (Baseline) | T2  (End-of-study) | Change  T2-T1 | Unadjusted between-group differences: T2 to T1 | Adjusted ^a^ between-group differences: T2 to T1 |
| --- | --- | --- | --- | --- | --- |
| Measure | Mean (SD) | Mean (SD) | Mean (SD) | Mean (95% CI) | Mean (95% CI) |
| Calorie  *(kcal/kg/d, n=144)*  Control  Heal-Me app | 1853 (696)  1737 (504) | 1814 (558)  1977 (689) | -39 (450)  240 (280) | 279 (83, 474) ^b^ | 246 (57,436) ^b^ |
| Protein  *(g/kg/d, n=144)*  Control  Heal-Me app | 86·6 (28·4)  78·7 (26·5) | 89·7 (30·0)  106·4 (37·0) | 3·1 (24·2)  27·6 (30·4) | 24·5 (14·2, 34·8) ^b^ | 22·8 (12·5, 33·1) ^b^ |
| BMI  *(kg/m^2^, n=155)*  Control  Heal-Me app | 28·9 (7·7)  29·1 (6·0) | 28·9 (7·4)  29·0 (6·0) | -0·6 (1·0)  0·0 (0·1) | -0·03 (-0·36, 0·30) | 0·04 (-0·30, 0·35) |

^a^ adjusted for age, Charlson comorbidity score, and baseline value

^b^ *p*<.05 between group improvement T2 to T1

**Figure S1.** The 2-minute step test mean percentage change for the control arm versus Heal-Me in the lowest and the highest quartiles.


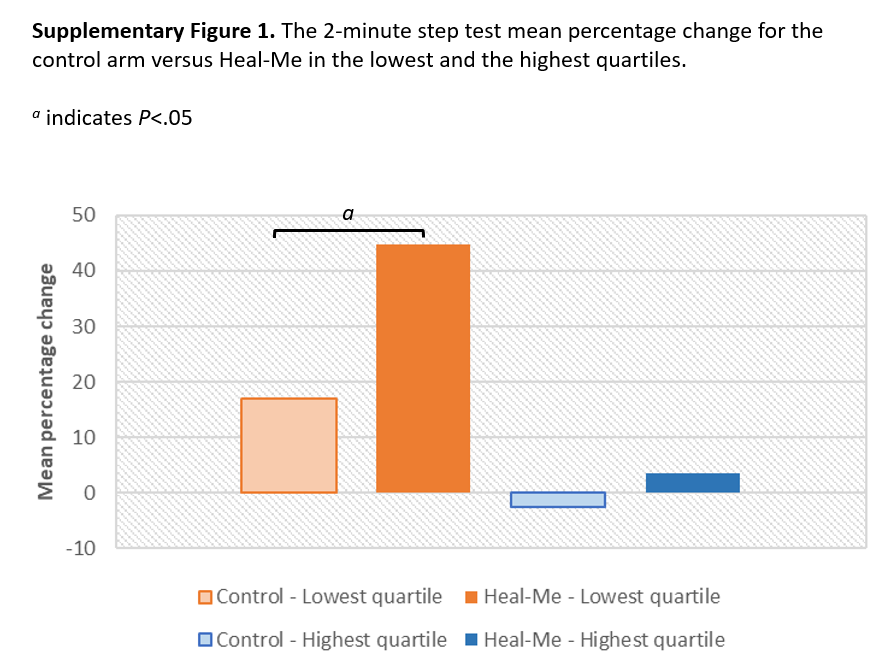


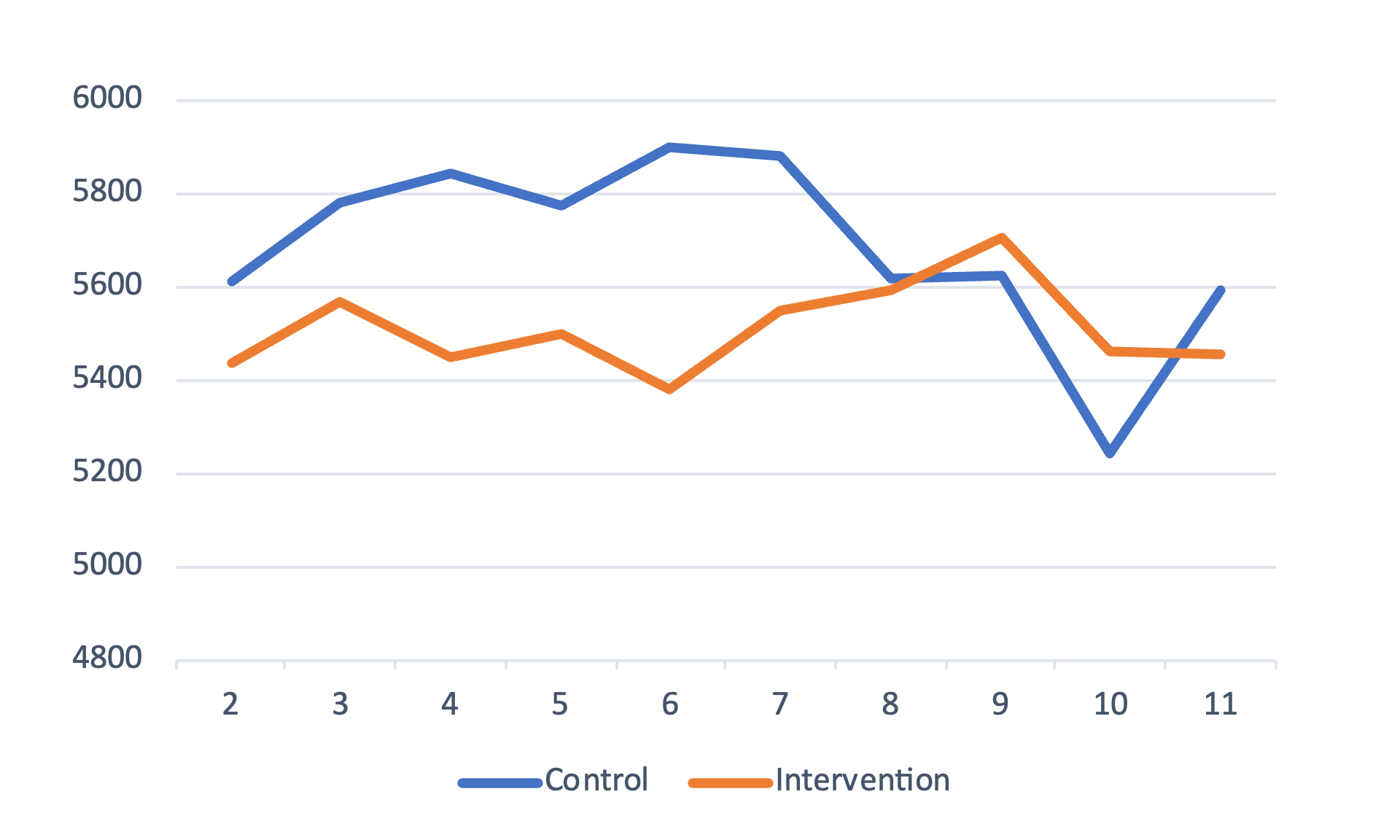


**Figure S2.** The mean number of step counts per week for the study weeks 2 to 11.


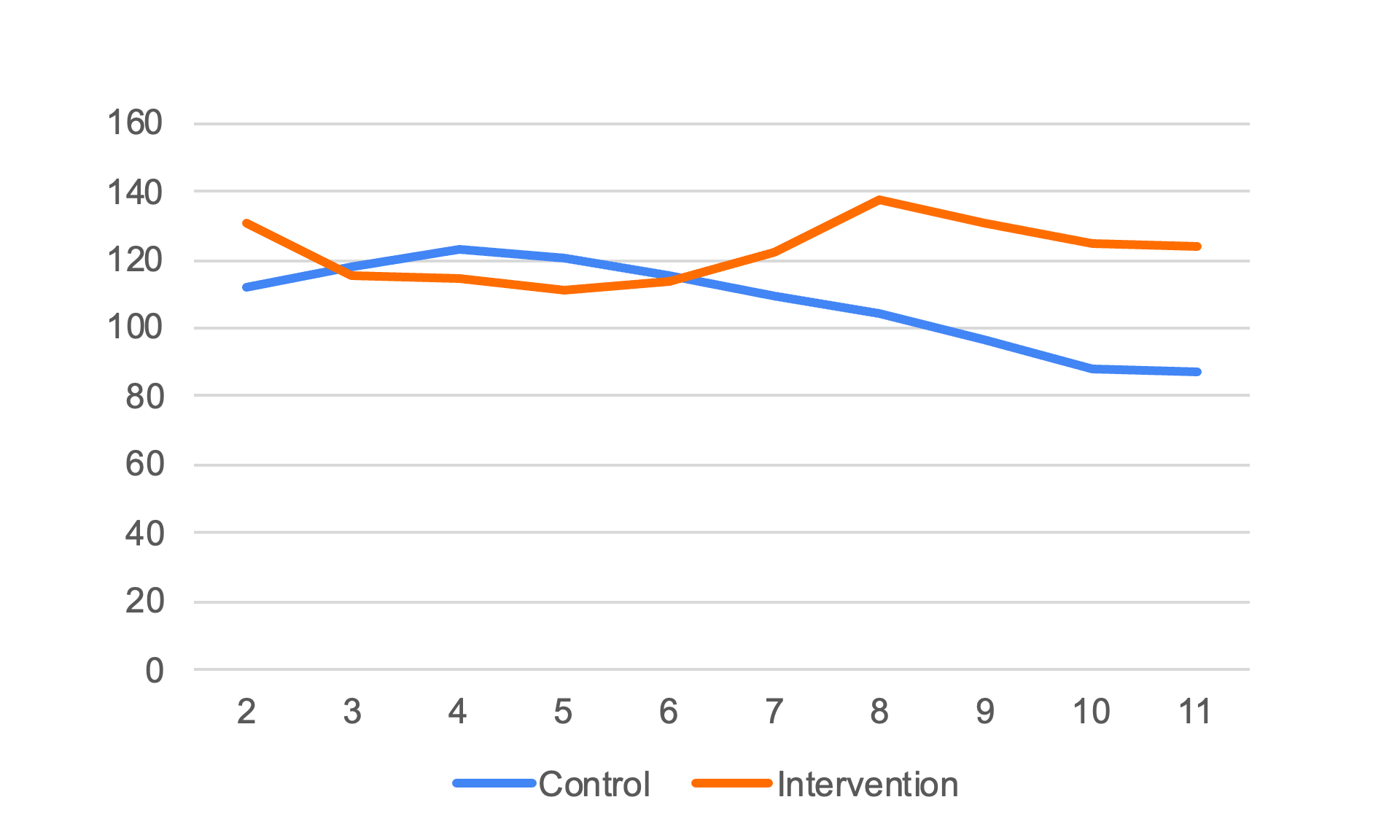


**Figure S3.** The mean number of minutes of moderate-to-vigorous physical activity per week for study weeks 2 to 11.
