# Supplementary material for: Acceptability and Effectiveness of a Fully Web-Based Nutrition and Exercise Program for Individuals With Chronic Disease During COVID-19: Randomized Controlled Trial
Source: J Med Internet Res. 2025 Mar 24;27:e57537. doi: 10.2196/57537 (PMC11976182; doi:10.2196/57537)
Supplement: Multimedia Appendix 2 [file jmir_v27i1e57537_app2.pdf]

CONSORT-EHEALTH (V 1.6.1) - Submission/Publication Form

The CONSORT-EHEALTH checklist is intended for authors of randomized trials evaluating web-based and Internet-based applications/interventions, including mobile interventions, electronic games (incl multiplayer games), social media, certain telehealth applications, and other interactive and/or networked electronic applications. Some of the items (e.g. all subitems under item 5 - description of the intervention) may also be applicable for other study designs.

The goal of the CONSORT EHEALTH checklist and guideline is to be
a) a guide for reporting for authors of RCTs,
b) to form a basis for appraisal of an ehealth trial (in terms of validity)

CONSORT-EHEALTH items/subitems are MANDATORY reporting items for studies published in the Journal of Medical Internet Research and other journals / scientific societies endorsing the checklist.

Items numbered 1., 2., 3., 4a., 4b etc are original CONSORT or CONSORT-NPT (non-pharmacologic treatment) items.
Items with Roman numerals (i., ii, iii, iv etc.) are CONSORT-EHEALTH extensions/clarifications.

As the CONSORT-EHEALTH checklist is still considered in a formative stage, we would ask that you also RATE ON A SCALE OF 1-5 how important/useful you feel each item is FOR THE PURPOSE OF THE CHECKLIST and reporting guideline (optional).

Mandatory reporting items are marked with a red *.
In the textboxes, either copy & paste the relevant sections from your manuscript into this form - please include any quotes from your manuscript in QUOTATION MARKS,
or answer directly by providing additional information not in the manuscript, or elaborating on why the item was not relevant for this study.

YOUR ANSWERS WILL BE PUBLISHED AS A SUPPLEMENTARY FILE TO YOUR PUBLICATION IN JMIR AND ARE CONSIDERED PART OF YOUR PUBLICATION (IF ACCEPTED).
Please fill in these questions diligently. Information will not be copyedited, so please use proper spelling and grammar, use correct capitalization, and avoid abbreviations.

DO NOT FORGET TO SAVE AS PDF _AND_ CLICK THE SUBMIT BUTTON SO YOUR ANSWERS ARE IN OUR DATABASE !!!

Citation Suggestion (if you append the pdf as Appendix we suggest to cite this paper in the caption):
Eysenbach G, CONSORT-EHEALTH Group

CONSORT-EHEALTH: Improving and Standardizing Evaluation Reports of Web-based and Mobile Health Interventions
J Med Internet Res 2011;13(4):e126
URL: http://www.jmir.org/2011/4/e126/
doi: 10.2196/jmir.1923
PMID: 22209829

kismond@ualberta.ca Switch account

Not shared

Draft saved

* Indicates required question

Your name *

First Last

Ismond

Primary Affiliation (short), City, Country *

University of Toronto, Toronto, Canada

University of Alberta, Edmonton, Canada

Your e-mail address *

abc@gmail.com

kismond@ualberta.ca

Title of your manuscript *

Provide the (draft) title of your manuscript.

Promise of a fully virtually delivered and evaluated web-based nutrition and exercise program for individuals with chronic disease: a randomized controlled trial during COVID-19

Name of your App/Software/Intervention *

If there is a short and a long/alternate name, write the short name first and add the long name in brackets.

Heal-Me

Evaluated Version (if any)

e.g. "V1", "Release 2017-03-01", "Version 2.0.27913"

Your answer

Language(s) *

What language is the intervention/app in? If multiple languages are available, separate by comma (e.g. "English, French")

English

URL of your Intervention Website or App

e.g. a direct link to the mobile app on app in appstore (itunes, Google Play), or URL of the website. If the intervention is a DVD or hardware, you can also link to an Amazon page.

Your answer

URL of an image/screenshot (optional)

Your answer

Accessibility *

Can an enduser access the intervention presently?

- ○ access is free and open
- ◉ access only for special usergroups, not open
- ○ access is open to everyone, but requires payment/subscription/in-app purchases
- ○ app/intervention no longer accessible
- ○ Other:

Primary Medical Indication/Disease/Condition *

e.g. "Stress", "Diabetes", or define the target group in brackets after the condition, e.g. "Autism (Parents of children with)", "Alzheimers (Informal Caregivers of)"

Chronic disease

Primary Outcomes measured in trial *

comma-separated list of primary outcomes reported in the trial

App acceptability (adherence & satisfaction) a

Secondary/other outcomes

Are there any other outcomes the intervention is expected to affect?

Secondary objective effectiveness outcomes included the 12-week change in objective physical function comparing the control and intervention groups for the: 2-minute step test, chair sit-to-stand (30 sec and 60 sec), plank, shoulder range of movement, and one-legged balance. Step-count and moderate-vigorous physical activity intensity minutes for weeks 2 to 11 were presented as a weekly average. Data from weeks 1 and 12 were not considered because set-up of the device often extended into the first week, and device return occurred at the end of week 12. Secondary self-reported effectiveness outcomes included UEFI-15, mental health, and quality of life (i.e., WHO-5 well-being index, GAD-7, DeJong Gierveld Loneliness Scale, SF-36, and the EQ-5D-5L). Adverse events were recorded by study staff.

Recommended "Dose" *

What do the instructions for users say on how often the app should be used?

- (●) Approximately Daily
- ( ) Approximately Weekly
- ( ) Approximately Monthly
- ( ) Approximately Yearly
- ( ) "as needed"
- ( ) Other:

Approx. Percentage of Users (starters) still using the app as recommended after 3 months *

- ○ unknown / not evaluated
- ○ 0-10%
- ○ 11-20%
- ○ 21-30%
- ○ 31-40%
- ○ 41-50%
- ○ 51-60%
- ○ 61-70%
- ○ 71%-80%
- ◉ 81-90%
- ○ 91-100%
- ○ Other:

Overall, was the app/intervention effective? *

- ○ yes: all primary outcomes were significantly better in intervention group vs control
- ◉ partly: SOME primary outcomes were significantly better in intervention group vs control
- ○ no statistically significant difference between control and intervention
- ○ potentially harmful: control was significantly better than intervention in one or more outcomes
- ○ inconclusive: more research is needed
- ○ Other:

Article Preparation Status/Stage *

At which stage in your article preparation are you currently (at the time you fill in this form)

- ○ not submitted yet - in early draft status
- ○ not submitted yet - in late draft status, just before submission
- ◉ submitted to a journal but not reviewed yet
- ○ submitted to a journal and after receiving initial reviewer comments
- ○ submitted to a journal and accepted, but not published yet
- ○ published
- ○ Other:

Journal *

If you already know where you will submit this paper (or if it is already submitted), please provide the journal name (if it is not JMIR, provide the journal name under "other")

- ○ not submitted yet / unclear where I will submit this
- ◉ Journal of Medical Internet Research (JMIR)
- ○ JMIR mHealth and UHealth
- ○ JMIR Serious Games
- ○ JMIR Mental Health
- ○ JMIR Public Health
- ○ JMIR Formative Research
- ○ Other JMIR sister journal
- ○ Other:

Is this a full powered effectiveness trial or a pilot/feasibility trial? *

- ( ) Pilot/feasibility
- (•) Fully powered

Manuscript tracking number *

If this is a JMIR submission, please provide the manuscript tracking number under "other" (The ms tracking number can be found in the submission acknowledgement email, or when you login as author in JMIR. If the paper is already published in JMIR, then the ms tracking number is the four-digit number at the end of the DOI, to be found at the bottom of each published article in JMIR)

- (•) no ms number (yet) / not (yet) submitted to / published in JMIR
- ( ) Other:

TITLE AND ABSTRACT

1a) TITLE: Identification as a randomized trial in the title

1a) Does your paper address CONSORT item 1a? *

I.e does the title contain the phrase "Randomized Controlled Trial"? (if not, explain the reason under "other")

- (•) yes
- ( ) Other:

1a-i) Identify the mode of delivery in the title

Identify the mode of delivery. Preferably use “web-based” and/or “mobile” and/or “electronic game” in the title. Avoid ambiguous terms like “online”, “virtual”, “interactive”. Use “Internet-based” only if Intervention includes non-web-based Internet components (e.g. email), use “computer-based” or “electronic” only if offline products are used. Use “virtual” only in the context of “virtual reality” (3-D worlds). Use “online” only in the context of “online support groups”. Complement or substitute product names with broader terms for the class of products (such as “mobile” or “smart phone” instead of “iphone”), especially if the application runs on different platforms.

	1	2	3	4	5	
subitem not at all important	○	○	◉	○	○	essential

Clear selection

Does your paper address subitem 1a-i? *

Copy and paste relevant sections from manuscript title (include quotes in quotation marks "like this" to indicate direct quotes from your manuscript), or elaborate on this item by providing additional information not in the ms, or briefly explain why the item is not applicable/relevant for your study

Promise of a fully virtually delivered and evaluated "web-based" nutrition and exercise program for individuals with chronic disease: a randomized controlled trial during COVID-19

1a-ii) Non-web-based components or important co-interventions in title

Mention non-web-based components or important co-interventions in title, if any (e.g., “with telephone support”).

	1	2	3	4	5	
subitem not at all important	○	◉	○	○	○	essential

Clear selection

Does your paper address subitem 1a-ii?

Copy and paste relevant sections from manuscript title (include quotes in quotation marks "like this" to indicate direct quotes from your manuscript), or elaborate on this item by providing additional information not in the ms, or briefly explain why the item is not applicable/relevant for your study

The Activity Tracker was a secondary outcome and was not essential to the study.

1a-iii) Primary condition or target group in the title

Mention primary condition or target group in the title, if any (e.g., "for children with Type I Diabetes") Example: A Web-based and Mobile Intervention with Telephone Support for Children with Type I Diabetes: Randomized Controlled Trial

	1	2	3	4	5	
subitem not at all important	○	○	○	○	◉	essential

Clear selection

Does your paper address subitem 1a-iii? *

Copy and paste relevant sections from manuscript title (include quotes in quotation marks "like this" to indicate direct quotes from your manuscript), or elaborate on this item by providing additional information not in the ms, or briefly explain why the item is not applicable/relevant for your study

Promise of a fully virtually delivered and evaluated web-based nutrition and exercise program for individuals with "chronic disease": a randomized controlled trial during COVID-19

1b) ABSTRACT: Structured summary of trial design, methods, results, and conclusions

NPT extension: Description of experimental treatment, comparator, care providers, centers, and blinding status.

1b-i) Key features/functionalities/components of the intervention and comparator in the METHODS section of the ABSTRACT

Mention key features/functionalities/components of the intervention and comparator in the abstract. If possible, also mention theories and principles used for designing the site. Keep in mind the needs of systematic reviewers and indexers by including important synonyms. (Note: Only report in the abstract what the main paper is reporting. If this information is missing from the main body of text, consider adding it)

	1	2	3	4	5	
subitem not at all important	○	○	○	◉	○	essential

Clear selection

Does your paper address subitem 1b-i? *

Copy and paste relevant sections from the manuscript abstract (include quotes in quotation marks "like this" to indicate direct quotes from your manuscript), or elaborate on this item by providing additional information not in the ms, or briefly explain why the item is not applicable/relevant for your study

Background
In-person nutrition and exercise interventions improve physical function in "chronic diseases". We report a semi-supervised nutrition and exercise program using a fully online process from recruitment through to end of trial measures in response to COVID-19.

Methods
Using a "3-arm randomized controlled trial design, we evaluated a 12-week program delivered across two levels of support as a web-based intervention ("Heal-Me"). Eligible adults (cancer, chronic lung disease, or transplant recipients) required access to an internet-connected device and had all previously completed exercise rehabilitation. Participants were randomized (1:1:1) to Arm 1 (self-directed online nutrition and exercise guide), Arm 2 (Heal-Me + dietitian/exercise specialist-led online group classes), or Arm 3 (Heal-Me + online group classes + one-to-one sessions)." All received a wearable activity tracker. Primary study objectives were acceptability and effectiveness. The primary effectiveness outcome was change in the self-reported Lower Extremity Functional Scale (LEFS) with secondary outcomes including physical function tests, and questionnaires for wellbeing, quality of life, and food intake. Analyses adhered to intention-to-treat.

1b-ii) Level of human involvement in the METHODS section of the ABSTRACT

Clarify the level of human involvement in the abstract, e.g., use phrases like “fully automated” vs. “therapist/nurse/care provider/physician-assisted” (mention number and expertise of providers involved, if any). (Note: Only report in the abstract what the main paper is reporting. If this information is missing from the main body of text, consider adding it)

	1	2	3	4	5	
subitem not at all important	○	○	○	○	◉	essential

Clear selection

Does your paper address subitem 1b-ii?

Copy and paste relevant sections from the manuscript abstract (include quotes in quotation marks "like this" to indicate direct quotes from your manuscript), or elaborate on this item by providing additional information not in the ms, or briefly explain why the item is not applicable/relevant for your study

"Participants were randomized (1:1:1) to Arm 1 (self-directed online nutrition and exercise guide), Arm 2 (Heal-Me + dietitian/exercise specialist-led online group classes), or Arm 3 (Heal-Me + online group classes + one-to-one sessions)."

1b-iii) Open vs. closed, web-based (self-assessment) vs. face-to-face assessments in the METHODS section of the ABSTRACT

Mention how participants were recruited (online vs. offline), e.g., from an open access website or from a clinic or a closed online user group (closed usergroup trial), and clarify if this was a purely web-based trial, or there were face-to-face components (as part of the intervention or for assessment). Clearly say if outcomes were self-assessed through questionnaires (as common in web-based trials). Note: In traditional offline trials, an open trial (open-label trial) is a type of clinical trial in which both the researchers and participants know which treatment is being administered. To avoid confusion, use "blinded" or "unblinded" to indicated the level of blinding instead of "open", as "open" in web-based trials usually refers to "open access" (i.e. participants can self-enrol). (Note: Only report in the abstract what the main paper is reporting. If this information is missing from the main body of text, consider adding it)

	1	2	3	4	5	
subitem not at all important	○	○	○	○	◉	essential

Clear selection

Does your paper address subitem 1b-iii?

Copy and paste relevant sections from the manuscript abstract (include quotes in quotation marks "like this" to indicate direct quotes from your manuscript), or elaborate on this item by providing additional information not in the ms, or briefly explain why the item is not applicable/relevant for your study

We report a semi-supervised nutrition and exercise program "using a fully online process from recruitment through to end of trial measures" in response to COVID-19.

1b-iv) RESULTS section in abstract must contain use data

Report number of participants enrolled/assessed in each group, the use/uptake of the intervention (e.g., attrition/adherence metrics, use over time, number of logins etc.), in addition to primary/secondary outcomes. (Note: Only report in the abstract what the main paper is reporting. If this information is missing from the main body of text, consider adding it)

	1	2	3	4	5	
subitem not at all important	○	○	○	○	◉	essential

Clear selection

Does your paper address subitem 1b-iv?

Copy and paste relevant sections from the manuscript abstract (include quotes in quotation marks "like this" to indicate direct quotes from your manuscript), or elaborate on this item by providing additional information not in the ms, or briefly explain why the item is not applicable/relevant for your study

"202 of 216 (94%) participants completed the intervention (mean: 61 ± 11 years; female: 64·4%; cancer: 62·4%; lung disease: 20·8%; lung transplant: 8·9%; liver transplant: 7·9%). Adherence exceeded a priori targets: 82% attended >75% of the program elements including post-intervention tests; and >80% reported moderate to high satisfaction. No significant differences were found for LEFS scores. Significant benefits were found in favor of Heal-Me supported programming for the change in 2-minute step test, WHO-5 well-being, scale SF-36 physical health and energy/fatigue scales, and target protein intake parameters. No serious adverse events occurred."

1b-v) CONCLUSIONS/DISCUSSION in abstract for negative trials

Conclusions/Discussions in abstract for negative trials: Discuss the primary outcome - if the trial is negative (primary outcome not changed), and the intervention was not used, discuss whether negative results are attributable to lack of uptake and discuss reasons. (Note: Only report in the abstract what the main paper is reporting. If this information is missing from the main body of text, consider adding it)

	1	2	3	4	5	
subitem not at all important	○	○	○	○	◉	essential

Clear selection

Does your paper address subitem 1b-v?

Copy and paste relevant sections from the manuscript abstract (include quotes in quotation marks "like this" to indicate direct quotes from your manuscript), or elaborate on this item by providing additional information not in the ms, or briefly explain why the item is not applicable/relevant for your study

"No significant differences were found for LEFS scores." AND "The demonstrated satisfaction, adherence, and effectiveness highlight the feasibility of a web-based semi-supervised nutrition and exercise intervention studied entirely online in individuals with chronic disease. The more intensive one-to-one interaction (Arm 3) led to greater improvements in perceived nutrition self-management."

INTRODUCTION

2a) In INTRODUCTION: Scientific background and explanation of rationale

2a-i) Problem and the type of system/solution

Describe the problem and the type of system/solution that is object of the study: intended as stand-alone intervention vs. incorporated in broader health care program? Intended for a particular patient population? Goals of the intervention, e.g., being more cost-effective to other interventions, replace or complement other solutions? (Note: Details about the intervention are provided in “Methods” under 5)

	1	2	3	4	5	
subitem not at all important	○	○	○	○	◉	essential

Clear selection

Does your paper address subitem 2a-i? *

Copy and paste relevant sections from the manuscript (include quotes in quotation marks "like this" to indicate direct quotes from your manuscript), or elaborate on this item by providing additional information not in the ms, or briefly explain why the item is not applicable/relevant for your study

"Internet-connected digital platforms, such as web-based applications (“apps”), while offering promise for the scalable delivery of multimodal supportive care interventions, have been associated with poor adherence and retention2. Moreover, little information was available to guide how much personnel support was needed to optimize app usage in older patient populations living with chronic disease." AND "To understand the extent of support needed by patient users, there were two intervention arms (online group classes only versus online group classes with additional one-to-one support). We hypothesized that increased contact with a dietitian and exercise professional would result in greater acceptability as measured by higher adherence to the program, and greater effectiveness as evaluated by change in the lower extremity function score (LEFS)."

2a-ii) Scientific background, rationale: What is known about the (type of) system

Scientific background, rationale: What is known about the (type of) system that is the object of the study (be sure to discuss the use of similar systems for other conditions/diagnoses, if appropiate), motivation for the study, i.e. what are the reasons for and what is the context for this specific study, from which stakeholder viewpoint is the study performed, potential impact of findings [2]. Briefly justify the choice of the comparator.

	1	2	3	4	5	
subitem not at all important	○	○	○	○	◉	essential

Clear selection

Does your paper address subitem 2a-ii? *

Copy and paste relevant sections from the manuscript (include quotes in quotation marks "like this" to indicate direct quotes from your manuscript), or elaborate on this item by providing additional information not in the ms, or briefly explain why the item is not applicable/relevant for your study

"At the pandemic's onset, limited data were available to guide the feasibility, safety, and effectiveness of delivering online nutrition and exercise therapy, and standardized protocols for conducting virtual evaluation of physical function were lacking. Internet-connected digital platforms, such as web-based applications ("apps"), while offering promise for the scalable delivery of multimodal supportive care interventions, have been associated with poor adherence and retention2. Moreover, little information was available to guide how much personnel support was needed to optimize app usage in older patient populations living with chronic disease. "

2b) In INTRODUCTION: Specific objectives or hypotheses

Does your paper address CONSORT subitem 2b? *

Copy and paste relevant sections from the manuscript (include quotes in quotation marks "like this" to indicate direct quotes from your manuscript), or elaborate on this item by providing additional information not in the ms, or briefly explain why the item is not applicable/relevant for your study

"We hypothesized that increased contact with a dietitian and exercise professional would result in greater acceptability as measured by higher adherence to the program, and greater effectiveness as evaluated by change in the lower extremity function score (LEFS)."

METHODS

3a) Description of trial design (such as parallel, factorial) including allocation ratio

Does your paper address CONSORT subitem 3a? *

Copy and paste relevant sections from the manuscript (include quotes in quotation marks "like this" to indicate direct quotes from your manuscript), or elaborate on this item by providing additional information not in the ms, or briefly explain why the item is not applicable/relevant for your study

"Personalized Online Nutrition and Exercise Routines (PiONEeR) was a parallel-group 3-arm RCT conducted using the Heal-Me web-based program across 12 weeks." AND "Allocation tables were generated, validated, and uploaded to the REDCap information system5 by an independent statistician. Stratification was based on chronic disease status: cancer, lung, or transplant. Randomization was a 1:1:1 ratio"

3b) Important changes to methods after trial commencement (such as eligibility criteria), with reasons

Does your paper address CONSORT subitem 3b? *

Copy and paste relevant sections from the manuscript (include quotes in quotation marks "like this" to indicate direct quotes from your manuscript), or elaborate on this item by providing additional information not in the ms, or briefly explain why the item is not applicable/relevant for your study

No changes were made once the trial began.

3b-i) Bug fixes, Downtimes, Content Changes

Bug fixes, Downtimes, Content Changes: ehealth systems are often dynamic systems. A description of changes to methods therefore also includes important changes made on the intervention or comparator during the trial (e.g., major bug fixes or changes in the functionality or content) (5-iii) and other "unexpected events" that may have influenced study design such as staff changes, system failures/downtimes, etc. [2].

	1	2	3	4	5	
subitem not at all important	◉	○	○	○	○	essential

Clear selection

Does your paper address subitem 3b-i?

Copy and paste relevant sections from the manuscript (include quotes in quotation marks "like this" to indicate direct quotes from your manuscript), or elaborate on this item by providing additional information not in the ms, or briefly explain why the item is not applicable/relevant for your study

This is not at all important because extensive usability and user experience testing had been conducted beforehand. During the trial, no notable bug fixes, downtimes, or content changes were made on the intervention or comparator during the trial.

4a) Eligibility criteria for participants

Does your paper address CONSORT subitem 4a? *

Copy and paste relevant sections from the manuscript (include quotes in quotation marks "like this" to indicate direct quotes from your manuscript), or elaborate on this item by providing additional information not in the ms, or briefly explain why the item is not applicable/relevant for your study

"Inclusion criteria included adults (age ≥ 18 years) with an internet-connected device in their home and the ability to read, write, and speak English. Individuals were eligible if they had cancer (completed an initial course of chemotherapy or radiotherapy but could be on maintenance therapy); had undergone a lung or liver transplant; or had chronic lung disease. All had to graduate from an exercise rehabilitation program before the COVID-19 public health restrictions. The requirement of being an exercise graduate reflected concerns over the safety and complexity of participants having to learn exercise in an entirely virtual environment on top of new tasks (e.g., wearable device, learning to use the Heal-Me app, nutrition tracking, and participating in home-based virtual physical function assessments) all during the time of COVID-19. Individuals were ineligible if they were receiving compassionate care with an anticipated survival <6 months, had unstable disease status, or were unable to provide informed electronic consent. "

4a-i) Computer / Internet literacy

Computer / Internet literacy is often an implicit “de facto” eligibility criterion - this should be explicitly clarified.

	1	2	3	4	5	
subitem not at all important	○	○	○	○	◉	essential

Clear selection

Does your paper address subitem 4a-i?

Copy and paste relevant sections from the manuscript (include quotes in quotation marks "like this" to indicate direct quotes from your manuscript), or elaborate on this item by providing additional information not in the ms, or briefly explain why the item is not applicable/relevant for your study

Device proficiency was measured to characterize the patient population. See Methods... "+), technology proficiency with an internet-connected device (either Computer Proficiency Questionnaire (CPQ) or Mobile Device Proficiency Questionnaire (MDPQ)),".

4a-ii) Open vs. closed, web-based vs. face-to-face assessments:

Open vs. closed, web-based vs. face-to-face assessments: Mention how participants were recruited (online vs. offline), e.g., from an open access website or from a clinic, and clarify if this was a purely web-based trial, or there were face-to-face components (as part of the intervention or for assessment), i.e., to what degree got the study team to know the participant. In online-only trials, clarify if participants were quasi-anonymous and whether having multiple identities was possible or whether technical or logistical measures (e.g., cookies, email confirmation, phone calls) were used to detect/prevent these.

	1	2	3	4	5	
subitem not at all important	○	○	○	○	◉	essential

Clear selection

Does your paper address subitem 4a-ii? *

Copy and paste relevant sections from the manuscript (include quotes in quotation marks "like this" to indicate direct quotes from your manuscript), or elaborate on this item by providing additional information not in the ms, or briefly explain why the item is not applicable/relevant for your study

"Study advertisement and recruitment were carried out by the virtual distribution of recruitment materials by frontline staff involved in oncology (Alberta Cancer Exercise, ACE), pulmonary (Breathe Easy), and post-transplant lung or liver rehabilitation programs (University of Alberta Hospitals) in Edmonton, Alberta. Interested participants were required to contact the research team for further information and eligibility screening. Participants were recruited and enrolled in cohorts that started monthly to facilitate group intervention sessions. "

4a-iii) Information giving during recruitment

Information given during recruitment. Specify how participants were briefed for recruitment and in the informed consent procedures (e.g., publish the informed consent documentation as appendix, see also item X26), as this information may have an effect on user self-selection, user expectation and may also bias results.

	1	2	3	4	5	
subitem not at all important	○	◉	○	○	○	essential

Clear selection

Does your paper address subitem 4a-iii?

Copy and paste relevant sections from the manuscript (include quotes in quotation marks "like this" to indicate direct quotes from your manuscript), or elaborate on this item by providing additional information not in the ms, or briefly explain why the item is not applicable/relevant for your study

"Study advertisement and recruitment were carried out by the virtual distribution of recruitment materials by frontline staff involved in oncology (Alberta Cancer Exercise, ACE), pulmonary (Breathe Easy), and post-transplant lung or liver rehabilitation programs (University of Alberta Hospitals) in Edmonton, Alberta. Interested participants were required to contact the research team for further information and eligibility screening. Participants were recruited and enrolled in cohorts that started monthly to facilitate group intervention sessions. "

4b) Settings and locations where the data were collected

Does your paper address CONSORT subitem 4b? *

Copy and paste relevant sections from the manuscript (include quotes in quotation marks "like this" to indicate direct quotes from your manuscript), or elaborate on this item by providing additional information not in the ms, or briefly explain why the item is not applicable/relevant for your study

"Thus, all trial stages, from recruitment through to end-of-study testing, were performed in a completely virtual environment, including participant technology training and the collection of data on physical function. "

4b-i) Report if outcomes were (self-)assessed through online questionnaires

Clearly report if outcomes were (self-)assessed through online questionnaires (as common in web-based trials) or otherwise.

	1	2	3	4	5	
subitem not at all important	○	○	○	◉	○	essential

Clear selection

Does your paper address subitem 4b-i? *

Copy and paste relevant sections from the manuscript (include quotes in quotation marks "like this" to indicate direct quotes from your manuscript), or elaborate on this item by providing additional information not in the ms, or briefly explain why the item is not applicable/relevant for your study

Yes, we indicated if the measure was self-reported or objectively measured as in the physical function measures done during video conferences. "Acceptability: This was comprised of adherence rate and satisfaction. Our primary acceptability outcome was adherence which was defined as completing >75% of the group nutrition and exercise sessions and completing all end of study end-of-study outcomes. Satisfaction was collected from the end of study program experience and satisfaction questionnaire. Two questions inquired about whether intervention participants felt prepared to continue their nutrition and exercise activities after program completion. Using a 5 item Likert scale (1, Not at all and 5, Very much), participants were asked: “Do you feel that the Pioneer nutrition program has prepared you to reach your target protein goal on your own?” and “Do you feel that the Pioneer exercise program has prepared you to reach your exercise goal on your own?” Technology acceptability was evaluated with the UTAUT and will be presented in a follow-on publication.

Effectiveness: This was comprised of a battery of objective outcomes including physical function tests and physical activity, and self-reported outcomes including quality of life. Our primary effectiveness outcome was the change in LEFS from baseline to 12 weeks between the control and combined intervention arms. Secondary objective effectiveness outcomes included the 12-week change in objective physical function comparing the control and intervention groups for the: 2-minute step test, chair sit-to-stand (30 sec and 60 sec), plank, shoulder range of movement, and one-legged balance. Step-count and moderate-vigorous physical activity intensity minutes for weeks 2 to 11 were presented as a weekly average. Data from weeks 1 and 12 were not considered because set-up of the device often extended into the first week, and device return occurred at the end of week 12. Secondary self-reported effectiveness outcomes included UEFI-15, mental health, and quality of life (i.e., WHO-5 well-being index, GAD-7, DeJong Gierveld Loneliness Scale, SF-36, and the EQ-5D-5L). Adverse events were recorded by study staff.

Exploratory outcomes (baseline and 12 weeks): Changes in daily calorie, protein intake, and the proportion of participants reaching target protein intake (>1.2 g/kg/day protein based on ideal body weight using a BMI of 24·9 kg/m2) were evaluated. The COM-B scale assessed changes in behavioral beliefs, with data planned for presentation in a future publication. For physical activity, participants were asked, “In the past week, on how many days have you done a total of 30 minutes or more of physical activity, which was enough to raise your breathing rate?10. Additionally, we explored results comparing the highest and lowest quartiles of baseline function for the 2-minute step test.
"

4b-ii) Report how institutional affiliations are displayed

Report how institutional affiliations are displayed to potential participants [on ehealth media], as affiliations with prestigious hospitals or universities may affect volunteer rates, use, and reactions with regards to an intervention.(Not a required item – describe only if this may bias results)

	1	2	3	4	5	
subitem not at all important	◉	○	○	○	○	essential

Clear selection

Does your paper address subitem 4b-ii?

Copy and paste relevant sections from the manuscript (include quotes in quotation marks "like this" to indicate direct quotes from your manuscript), or elaborate on this item by providing additional information not in the ms, or briefly explain why the item is not applicable/relevant for your study

The University of Alberta was displayed on all recruitment material. Funders for the Heal-Me app are listed under the About section.

5) The interventions for each group with sufficient details to allow replication, including how and when they were actually administered

5-i) Mention names, credential, affiliations of the developers, sponsors, and owners

Mention names, credential, affiliations of the developers, sponsors, and owners [6] (if authors/evaluators are owners or developer of the software, this needs to be declared in a “Conflict of interest” section or mentioned elsewhere in the manuscript).

	1	2	3	4	5	
subitem not at all important	◉	○	○	○	○	essential

Clear selection

Does your paper address subitem 5-i?

Copy and paste relevant sections from the manuscript (include quotes in quotation marks "like this" to indicate direct quotes from your manuscript), or elaborate on this item by providing additional information not in the ms, or briefly explain why the item is not applicable/relevant for your study

No. There are no conflicts of interest. The app is not a commercial entity nor is it planned to become one. It is a research tool. Currently it is being used in research studies led by other investigators at other institutions.

5-ii) Describe the history/development process

Describe the history/development process of the application and previous formative evaluations (e.g., focus groups, usability testing), as these will have an impact on adoption/use rates and help with interpreting results.

	1	2	3	4	5	
subitem not at all important	○	○	○	○	◉	essential

Clear selection

Does your paper address subitem 5-ii?

Copy and paste relevant sections from the manuscript (include quotes in quotation marks "like this" to indicate direct quotes from your manuscript), or elaborate on this item by providing additional information not in the ms, or briefly explain why the item is not applicable/relevant for your study

The content is include in the protocol paper. The protocol paper is cited frequently throughout the current article reporting the results of the RCT... For example: "Personalized Online Nutrition and Exercise Routines (PiONEeR) was a parallel-group 3-arm RCT conducted using the Heal-Me web-based program across 12 weeks (4)." Citation 4 is the protocol paper published in 2022.

5-iii) Revisions and updating

Revisions and updating. Clearly mention the date and/or version number of the application/intervention (and comparator, if applicable) evaluated, or describe whether the intervention underwent major changes during the evaluation process, or whether the development and/or content was “frozen” during the trial. Describe dynamic components such as news feeds or changing content which may have an impact on the replicability of the intervention (for unexpected events see item 3b).

	1	2	3	4	5	
subitem not at all important	(●)	( )	( )	( )	( )	essential

Clear selection

Does your paper address subitem 5-iii?

Copy and paste relevant sections from the manuscript (include quotes in quotation marks "like this" to indicate direct quotes from your manuscript), or elaborate on this item by providing additional information not in the ms, or briefly explain why the item is not applicable/relevant for your study

There were no revisions or updating between the protocol paper, the beginning of the RCT, or the end of the RCT. Hence, this is not explicitly mentioned.

5-iv) Quality assurance methods

Provide information on quality assurance methods to ensure accuracy and quality of information provided [1], if applicable.

	1	2	3	4	5	
subitem not at all important	(●)	( )	( )	( )	( )	essential

Clear selection

Does your paper address subitem 5-iv?

Copy and paste relevant sections from the manuscript (include quotes in quotation marks "like this" to indicate direct quotes from your manuscript), or elaborate on this item by providing additional information not in the ms, or briefly explain why the item is not applicable/relevant for your study

The nutrition and exercise knowledge was evidence-based from the most recent disease-specific guidelines. Interactions with the staff members were scripted including the app walkthrough/training session to ensure consistency in material delivery. This information was included in the study protocol published in 2022.

5-v) Ensure replicability by publishing the source code, and/or providing screenshots/screen-capture video, and/or providing flowcharts of the algorithms used

Ensure replicability by publishing the source code, and/or providing screenshots/screen-capture video, and/or providing flowcharts of the algorithms used. Replicability (i.e., other researchers should in principle be able to replicate the study) is a hallmark of scientific reporting.

	1	2	3	4	5	
subitem not at all important	(●)	( )	( )	( )	( )	essential

Clear selection

Does your paper address subitem 5-v?

Copy and paste relevant sections from the manuscript (include quotes in quotation marks "like this" to indicate direct quotes from your manuscript), or elaborate on this item by providing additional information not in the ms, or briefly explain why the item is not applicable/relevant for your study

This information was presented in the protocol paper describing the web-based app Heal-Me.

5-vi) Digital preservation

Digital preservation: Provide the URL of the application, but as the intervention is likely to change or disappear over the course of the years; also make sure the intervention is archived (Internet Archive, webcitation.org, and/or publishing the source code or screenshots/videos alongside the article). As pages behind login screens cannot be archived, consider creating demo pages which are accessible without login.

	1	2	3	4	5	
subitem not at all important	(●)	○	○	○	○	essential

Clear selection

Does your paper address subitem 5-vi?

Copy and paste relevant sections from the manuscript (include quotes in quotation marks "like this" to indicate direct quotes from your manuscript), or elaborate on this item by providing additional information not in the ms, or briefly explain why the item is not applicable/relevant for your study

No it does not. The reasons for this is that the app has been in redevelopment since the completion of the RCT to add features and functions to improve the user experience.

5-vii) Access

Access: Describe how participants accessed the application, in what setting/context, if they had to pay (or were paid) or not, whether they had to be a member of specific group. If known, describe how participants obtained "access to the platform and Internet" [1]. To ensure access for editors/reviewers/readers, consider to provide a "backdoor" login account or demo mode for reviewers/readers to explore the application (also important for archiving purposes, see vi).

	1	2	3	4	5	
subitem not at all important	○	○	○	(●)	○	essential

Clear selection

Does your paper address subitem 5-vii? *

Copy and paste relevant sections from the manuscript (include quotes in quotation marks "like this" to indicate direct quotes from your manuscript), or elaborate on this item by providing additional information not in the ms, or briefly explain why the item is not applicable/relevant for your study

Participants had to be randomized to an intervention group and then were provided with a weblink and login materials. "Participants in the intervention Arms 2 and 3 had three virtual sessions detailed in the protocol4: (1) an introduction to and instruction in the use of the Heal-Me app; [including a reference to the protocol paper]"

5-viii) Mode of delivery, features/functionalities/components of the intervention and comparator, and the theoretical framework

Describe mode of delivery, features/functionalities/components of the intervention and comparator, and the theoretical framework [6] used to design them (instructional strategy [1], behaviour change techniques, persuasive features, etc., see e.g., [7, 8] for terminology). This includes an in-depth description of the content (including where it is coming from and who developed it) [1],” whether [and how] it is tailored to individual circumstances and allows users to track their progress and receive feedback” [6]. This also includes a description of communication delivery channels and – if computer-mediated communication is a component – whether communication was synchronous or asynchronous [6]. It also includes information on presentation strategies [1], including page design principles, average amount of text on pages, presence of hyperlinks to other resources, etc. [1].

	1	2	3	4	5	
subitem not at all important	○	○	○	○	◉	essential

Clear selection

Does your paper address subitem 5-viii? *

Copy and paste relevant sections from the manuscript (include quotes in quotation marks "like this" to indicate direct quotes from your manuscript), or elaborate on this item by providing additional information not in the ms, or briefly explain why the item is not applicable/relevant for your study

"Version 1 of the app was used in the current study and is described in more detail in the protocol paper which includes illustrations of user interface and app features4. The Heal-Me web-based app facilitates semi-supervised nutrition and exercise programming tailorable to the unique needs of each patient user. It includes nutrition content - a daily protein tracking system, high protein recipes, and cooking videos; exercise content - customizable follow-along exercise videos and auto-tracking of videos watched; and a calendar for scheduling and accessing online group classes and one-to-one check-ins. To guide behavior change, the capability, opportunity, and motivation behavior (COM-B) model and the theoretical domains framework (TDF) were employed as part of the development of the Heal-Me intervention to inform progress tracking, provide demonstration videos, and graded tasks4."

5-ix) Describe use parameters

Describe use parameters (e.g., intended “doses” and optimal timing for use). Clarify what instructions or recommendations were given to the user, e.g., regarding timing, frequency, heaviness of use, if any, or was the intervention used ad libitum.

	1	2	3	4	5	
subitem not at all important	○	○	○	○	◉	essential

Clear selection

Does your paper address subitem 5-ix?

Copy and paste relevant sections from the manuscript (include quotes in quotation marks "like this" to indicate direct quotes from your manuscript), or elaborate on this item by providing additional information not in the ms, or briefly explain why the item is not applicable/relevant for your study

"Participants were asked to complete 45-60 minutes of exercise sessions three times a week (two independent sessions, one CES-led online group class), protein tracking for three days each week, and attending five online group nutrition classes. Arm 3 (Personnel intensive), in addition, received one-to-one check-ins with the dietitian (2x, 20-30 minutes) and CES (5x, 20-30 minutes) over the 12-week study period. "

5-x) Clarify the level of human involvement

Clarify the level of human involvement (care providers or health professionals, also technical assistance) in the e-intervention or as co-intervention (detail number and expertise of professionals involved, if any, as well as “type of assistance offered, the timing and frequency of the support, how it is initiated, and the medium by which the assistance is delivered”. It may be necessary to distinguish between the level of human involvement required for the trial, and the level of human involvement required for a routine application outside of a RCT setting (discuss under item 21 – generalizability).

	1	2	3	4	5	
subitem not at all important	○	○	○	○	◉	essential

Clear selection

Does your paper address subitem 5-x?

Copy and paste relevant sections from the manuscript (include quotes in quotation marks "like this" to indicate direct quotes from your manuscript), or elaborate on this item by providing additional information not in the ms, or briefly explain why the item is not applicable/relevant for your study

"Participants were randomized and allocated to one of three study arms after completing the baseline measures. All participants received a wearable activity tracker (Garmin Ltd., Olathe, KS) to monitor daily step counts, moderate-vigorous physical activity intensity minutes, and online assistance with set-up. The self-directed control group received a 52-page manual containing illustrated exercise routines at three levels and nutrition guidance (Alberta Health Services and Canada's Food Guide).

Participants in the intervention Arms 2 and 3 had three virtual sessions detailed in the protocol4: (1) an introduction to and instruction in the use of the Heal-Me app; (2) a dietitian-led nutrition assessment, review of nutrition preferences, setting of a protein goal target in the app (1·2-1·5 g/kg/day based on guidelines7-9). While the focus of the nutrition counseling was on protein intake, a condition specific guideline based target for daily caloric intake of 25-30 kcal/kg/day was also presented in the app, and counseling was provided about eating a balanced plate7-9. Lastly, (3) a CES-led review of home space for exercise, review of exercise preferences, and setting up of an individualized program in the app. Recognizing the limited data supporting safety of virtual exercise, the CES started all participants at a low intensity level for exercise"

5-xi) Report any prompts/reminders used

Report any prompts/reminders used: Clarify if there were prompts (letters, emails, phone calls, SMS) to use the application, what triggered them, frequency etc. It may be necessary to distinguish between the level of prompts/reminders required for the trial, and the level of prompts/reminders for a routine application outside of a RCT setting (discuss under item 21 – generalizability).

	1	2	3	4	5	
subitem not at all important	○	○	○	○	◉	essential

Clear selection

Does your paper address subitem 5-xi? *

Copy and paste relevant sections from the manuscript (include quotes in quotation marks "like this" to indicate direct quotes from your manuscript), or elaborate on this item by providing additional information not in the ms, or briefly explain why the item is not applicable/relevant for your study

While important ot the delivery of the app and adherence, this information was included in the protocol paper published in 2022.

5-xii) Describe any co-interventions (incl. training/support)

Describe any co-interventions (incl. training/support): Clearly state any interventions that are provided in addition to the targeted eHealth intervention, as ehealth intervention may not be designed as stand-alone intervention. This includes training sessions and support [1]. It may be necessary to distinguish between the level of training required for the trial, and the level of training for a routine application outside of a RCT setting (discuss under item 21 – generalizability.

	1	2	3	4	5	
subitem not at all important	○	○	○	○	◉	essential

Clear selection

Does your paper address subitem 5-xii? *

Copy and paste relevant sections from the manuscript (include quotes in quotation marks "like this" to indicate direct quotes from your manuscript), or elaborate on this item by providing additional information not in the ms, or briefly explain why the item is not applicable/relevant for your study

"Participants in the intervention Arms 2 and 3 had three virtual sessions detailed in the protocol4: (1) an introduction to and instruction in the use of the Heal-Me app; (2) a dietitian-led nutrition assessment, review of nutrition preferences, setting of a protein goal target in the app (1·2-1·5 g/kg/day based on guidelines7-9). While the focus of the nutrition counseling was on protein intake, a condition specific guideline based target for daily caloric intake of 25-30 kcal/kg/day was also presented in the app, and counseling was provided about eating a balanced plate7-9. Lastly, (3) a CES-led review of home space for exercise, review of exercise preferences, and setting up of an individualized program in the app."

6a) Completely defined pre-specified primary and secondary outcome measures, including how and when they were assessed

Does your paper address CONSORT subitem 6a? *

Copy and paste relevant sections from the manuscript (include quotes in quotation marks "like this" to indicate direct quotes from your manuscript), or elaborate on this item by providing additional information not in the ms, or briefly explain why the item is not applicable/relevant for your study

"Acceptability: This was comprised of adherence rate and satisfaction. Our primary acceptability outcome was adherence which was defined as completing >75% of the group nutrition and exercise sessions and completing all end of study end-of-study outcomes. Satisfaction was collected from the end of study program experience and satisfaction questionnaire. Two questions inquired about whether intervention participants felt prepared to continue their nutrition and exercise activities after program completion. Using a 5 item Likert scale (1, Not at all and 5, Very much), participants were asked: “Do you feel that the Pioneer nutrition program has prepared you to reach your target protein goal on your own?” and “Do you feel that the Pioneer exercise program has prepared you to reach your exercise goal on your own?” Technology acceptability was evaluated with the UTAUT and will be presented in a follow-on publication.

Effectiveness: This was comprised of a battery of objective outcomes including physical function tests and physical activity, and self-reported outcomes including quality of life. Our primary effectiveness outcome was the change in LEFS from baseline to 12 weeks between the control and combined intervention arms. Secondary objective effectiveness outcomes included the 12-week change in objective physical function comparing the control and intervention groups for the: 2-minute step test, chair sit-to-stand (30 sec and 60 sec), plank, shoulder range of movement, and one-legged balance. Step-count and moderate-vigorous physical activity intensity minutes for weeks 2 to 11 were presented as a weekly average. Data from weeks 1 and 12 were not considered because set-up of the device often extended into the first week, and device return occurred at the end of week 12. Secondary self-reported effectiveness outcomes included UEFI-15, mental health, and quality of life (i.e., WHO-5 well-being index, GAD-7, DeJong Gierveld Loneliness Scale, SF-36, and the EQ-5D-5L). Adverse events were recorded by study staff.

Exploratory outcomes (baseline and 12 weeks): Changes in daily calorie, protein intake, and the proportion of participants reaching target protein intake (>1.2 g/kg/day protein based on ideal body weight using a BMI of 24·9 kg/m2) were evaluated. The COM-B scale assessed changes in behavioral beliefs, with data planned for presentation in a future publication. For physical activity, participants were asked, “In the past week, on how many days have you done a total of 30 minutes or more of physical activity, which was enough to raise your breathing rate?10. Additionally, we explored results comparing the highest and lowest quartiles of baseline function for the 2-minute step test. "

6a-i) Online questionnaires: describe if they were validated for online use and apply CHERRIES items to describe how the questionnaires were designed/deployed

If outcomes were obtained through online questionnaires, describe if they were validated for online use and apply CHERRIES items to describe how the questionnaires were designed/deployed [9].

	1	2	3	4	5	
subitem not at all important	○	○	○	○	◉	essential

Clear selection

Does your paper address subitem 6a-i?

Copy and paste relevant sections from manuscript text

Consistent with the CHERRIES checklist, all questionnaires were administered through the research system REDCap. The system itself has been approved for use in research settings and fufills the CHERRIES checklist requirement for data handling in the virtual space. All questionnaires used had been previously validated in paper-based formats.

6a-ii) Describe whether and how "use" (including intensity of use/dosage) was defined/measured/monitored

Describe whether and how "use" (including intensity of use/dosage) was defined/measured/monitored (logins, logfile analysis, etc.). Use/adoption metrics are important process outcomes that should be reported in any ehealth trial.

	1	2	3	4	5	
subitem not at all important	○	○	○	○	◉	essential

Clear selection

Does your paper address subitem 6a-ii?

Copy and paste relevant sections from manuscript text

Intensity of use/dosages was informed by the guidelines on nutrition and exercise for each specific disease condition. The Canadian Health Food Guide and Exercise Guide were also consulted. Details are included in the protocol paper.

6a-iii) Describe whether, how, and when qualitative feedback from participants was obtained

Describe whether, how, and when qualitative feedback from participants was obtained (e.g., through emails, feedback forms, interviews, focus groups).

	1	2	3	4	5	
subitem not at all important	◉	○	○	○	○	essential

Clear selection

Does your paper address subitem 6a-iii?

Copy and paste relevant sections from manuscript text

There are no qualitative feedback data collected in this study.

6b) Any changes to trial outcomes after the trial commenced, with reasons

Does your paper address CONSORT subitem 6b? *

Copy and paste relevant sections from the manuscript (include quotes in quotation marks "like this" to indicate direct quotes from your manuscript), or elaborate on this item by providing additional information not in the ms, or briefly explain why the item is not applicable/relevant for your study

There were no changes to the trial outcomes after the trial commenced.

7a) How sample size was determined

NPT: When applicable, details of whether and how the clustering by care provides or centers was addressed

7a-i) Describe whether and how expected attrition was taken into account when calculating the sample size

Describe whether and how expected attrition was taken into account when calculating the sample size.

	1	2	3	4	5	
subitem not at all important	○	○	○	○	●	essential

Clear selection

Does your paper address subitem 7a-i?

Copy and paste relevant sections from manuscript title (include quotes in quotation marks "like this" to indicate direct quotes from your manuscript), or elaborate on this item by providing additional information not in the ms, or briefly explain why the item is not applicable/relevant for your study

"To account for a 20% non-completion rate, the total sample size was increased to 216 participants. "

7b) When applicable, explanation of any interim analyses and stopping guidelines

Does your paper address CONSORT subitem 7b? *

Copy and paste relevant sections from the manuscript (include quotes in quotation marks "like this" to indicate direct quotes from your manuscript), or elaborate on this item by providing additional information not in the ms, or briefly explain why the item is not applicable/relevant for your study

There were no interim analyses or stopping guidelines.

8a) Method used to generate the random allocation sequence

NPT: When applicable, how care providers were allocated to each trial group

Does your paper address CONSORT subitem 8a? *

Copy and paste relevant sections from the manuscript (include quotes in quotation marks "like this" to indicate direct quotes from your manuscript), or elaborate on this item by providing additional information not in the ms, or briefly explain why the item is not applicable/relevant for your study

"Allocation tables were generated, validated, and uploaded to the REDCap information system5 by an independent statistician. Stratification was based on chronic disease status: cancer, lung, or transplant. Randomization was a 1:1:1 ratio:

- Arm 1, control (self-directed, online nutrition and exercise guide)
- Arm 2, personnel light (Heal-Me app and online group sessions led by Heal-Me trainers – trainers defined as a registered dietitian (RD) and certified exercise specialists (CES) including a physiotherapist)
- Arm 3, personnel intensive (Heal-Me app, online group sessions, and one-to-one meetings with trainers).

Allocation was concealed from all study staff. The end-of-trial independent assessor was blinded to the participants' group allocation. "

8b) Type of randomisation; details of any restriction (such as blocking and block size)

Does your paper address CONSORT subitem 8b? *

Copy and paste relevant sections from the manuscript (include quotes in quotation marks "like this" to indicate direct quotes from your manuscript), or elaborate on this item by providing additional information not in the ms, or briefly explain why the item is not applicable/relevant for your study

No randomization restrictions were implemented.

9) Mechanism used to implement the random allocation sequence (such as sequentially numbered containers), describing any steps taken to conceal the sequence until interventions were assigned

Does your paper address CONSORT subitem 9? *

Copy and paste relevant sections from the manuscript (include quotes in quotation marks "like this" to indicate direct quotes from your manuscript), or elaborate on this item by providing additional information not in the ms, or briefly explain why the item is not applicable/relevant for your study

"Allocation tables were generated, validated, and uploaded to the REDCap information system5 by an independent statistician. Stratification was based on chronic disease status: cancer, lung, or transplant. Randomization was a 1:1:1 ratio:

- Arm 1, control (self-directed, online nutrition and exercise guide)
- Arm 2, personnel light (Heal-Me app and online group sessions led by Heal-Me trainers – trainers defined as a registered dietitian (RD) and certified exercise specialists (CES) including a physiotherapist)
- Arm 3, personnel intensive (Heal-Me app, online group sessions, and one-to-one meetings with trainers).

Allocation was concealed from all study staff. The end-of-trial independent assessor was blinded to the participants' group allocation. "

10) Who generated the random allocation sequence, who enrolled participants, and who assigned participants to interventions

Does your paper address CONSORT subitem 10? *

Copy and paste relevant sections from the manuscript (include quotes in quotation marks "like this" to indicate direct quotes from your manuscript), or elaborate on this item by providing additional information not in the ms, or briefly explain why the item is not applicable/relevant for your study

Allocation tables were generated, validated, and uploaded to the REDCap information system5 by "an independent statistician."

11a) If done, who was blinded after assignment to interventions (for example, participants, care providers, those assessing outcomes) and how

NPT: Whether or not administering co-interventions were blinded to group assignment

11a-i) Specify who was blinded, and who wasn't

Specify who was blinded, and who wasn't. Usually, in web-based trials it is not possible to blind the participants [1, 3] (this should be clearly acknowledged), but it may be possible to blind outcome assessors, those doing data analysis or those administering co-interventions (if any).

	1	2	3	4	5	
subitem not at all important	○	○	○	○	◉	essential

Clear selection

Does your paper address subitem 11a-i? *

Copy and paste relevant sections from the manuscript (include quotes in quotation marks "like this" to indicate direct quotes from your manuscript), or elaborate on this item by providing additional information not in the ms, or briefly explain why the item is not applicable/relevant for your study

"The end-of-trial independent assessor was blinded to the participants' group allocation. "

11a-ii) Discuss e.g., whether participants knew which intervention was the "intervention of interest" and which one was the "comparator"

Informed consent procedures (4a-ii) can create biases and certain expectations - discuss e.g., whether participants knew which intervention was the "intervention of interest" and which one was the "comparator".

	1	2	3	4	5	
subitem not at all important	○	○	○	○	◉	essential

Clear selection

Does your paper address subitem 11a-ii?

Copy and paste relevant sections from the manuscript (include quotes in quotation marks "like this" to indicate direct quotes from your manuscript), or elaborate on this item by providing additional information not in the ms, or briefly explain why the item is not applicable/relevant for your study

Because recruitment, randomization, and baseline measures were performed virtually, participants had not opportunity to interact with one another and thus were blinded to the allocation between the control and the intervention groups. The participants in the two intervention groups were likely not blinded fully as they were a mixed group in the online nutrition and group exercise sessions. There was no forum or opportunity for participants to communicate with one another within the web-based app.

11b) If relevant, description of the similarity of interventions

(this item is usually not relevant for ehealth trials as it refers to similarity of a placebo or sham intervention to a active medication/intervention)

Does your paper address CONSORT subitem 11b? *

Copy and paste relevant sections from the manuscript (include quotes in quotation marks "like this" to indicate direct quotes from your manuscript), or elaborate on this item by providing additional information not in the ms, or briefly explain why the item is not applicable/relevant for your study

'• Arm 1, control (self-directed, online nutrition and exercise guide)
• Arm 2, personnel light (Heal-Me app and online group sessions led by Heal-Me trainers – trainers defined as a registered dietitian (RD) and certified exercise specialists (CES) including a physiotherapist)
• Arm 3, personnel intensive (Heal-Me app, online group sessions, and one-to-one meetings with trainers). "

12a) Statistical methods used to compare groups for primary and secondary outcomes

NPT: When applicable, details of whether and how the clustering by care providers or centers was addressed

Does your paper address CONSORT subitem 12a? *

Copy and paste relevant sections from the manuscript (include quotes in quotation marks "like this" to indicate direct quotes from your manuscript), or elaborate on this item by providing additional information not in the ms, or briefly explain why the item is not applicable/relevant for your study

"The intention-to-treat (ITT) principle was used for all analyses. Descriptive statistics were used to understand the participants' characteristics and outcome variables. The change between groups was analyzed using ANOVA (or its nonparametric alternative) and Tukey HSD test for pairwise comparisons using a family-wise alpha of 0·05. The primary analysis compared the difference in LEFS score between week 12 and study baseline between the combined app arms versus control. Three-group comparison was planned only if the combined two-group comparison was significant. The differences in the frequency of adverse events were assessed using the chi-square test. Linear regressions were constructed to determine clinically significant outcomes, adjusted for sex, age, and comorbidity status; group assignment was an independent variable"

12a-i) Imputation techniques to deal with attrition / missing values

Imputation techniques to deal with attrition / missing values: Not all participants will use the intervention/comparator as intended and attrition is typically high in ehealth trials. Specify how participants who did not use the application or dropped out from the trial were treated in the statistical analysis (a complete case analysis is strongly discouraged, and simple imputation techniques such as LOCF may also be problematic [4]).

	1	2	3	4	5	
subitem not at all important	○	○	◉	○	○	essential

Clear selection

Does your paper address subitem 12a-i? *

Copy and paste relevant sections from the manuscript (include quotes in quotation marks "like this" to indicate direct quotes from your manuscript), or elaborate on this item by providing additional information not in the ms, or briefly explain why the item is not applicable/relevant for your study

Missing values were omitted from the final analyses. Our prior pilot work had low levels of attrition, so we were optimistic.

12b) Methods for additional analyses, such as subgroup analyses and adjusted analyses

Does your paper address CONSORT subitem 12b? *

Copy and paste relevant sections from the manuscript (include quotes in quotation marks "like this" to indicate direct quotes from your manuscript), or elaborate on this item by providing additional information not in the ms, or briefly explain why the item is not applicable/relevant for your study

"Three-group comparison was planned only if the combined two-group comparison was significant."

X26) REB/IRB Approval and Ethical Considerations [recommended as subheading under "Methods"] (not a CONSORT item)

X26-i) Comment on ethics committee approval

	1	2	3	4	5	
subitem not at all important	○	○	○	○	●	essential

Clear selection

Does your paper address subitem X26-i?

Copy and paste relevant sections from the manuscript (include quotes in quotation marks "like this" to indicate direct quotes from your manuscript), or elaborate on this item by providing additional information not in the ms, or briefly explain why the item is not applicable/relevant for your study

"The study was approved by the University of Alberta's Health Research Ethics Board (Pro00103715) and was registered with clinicaltrials.gov (NCT04666558)."

x26-ii) Outline informed consent procedures

Outline informed consent procedures e.g., if consent was obtained offline or online (how? Checkbox, etc.?), and what information was provided (see 4a-ii). See [6] for some items to be included in informed consent documents.

	1	2	3	4	5	
subitem not at all important	●	○	○	○	○	essential

Clear selection

Does your paper address subitem X26-ii?

Copy and paste relevant sections from the manuscript (include quotes in quotation marks "like this" to indicate direct quotes from your manuscript), or elaborate on this item by providing additional information not in the ms, or briefly explain why the item is not applicable/relevant for your study

As stated as a fully online trial from recruitment to end of study measures, the informed consent was collected online through the REDCap research system.

X26-iii) Safety and security procedures

Safety and security procedures, incl. privacy considerations, and any steps taken to reduce the likelihood or detection of harm (e.g., education and training, availability of a hotline)

	1	2	3	4	5	
subitem not at all important	○	○	◉	○	○	essential

Clear selection

Does your paper address subitem X26-iii?

Copy and paste relevant sections from the manuscript (include quotes in quotation marks "like this" to indicate direct quotes from your manuscript), or elaborate on this item by providing additional information not in the ms, or briefly explain why the item is not applicable/relevant for your study

Participants could not communicate with one another through the web-based app. During online group sessions, participants had the option to turn their cameras on or off at their discretion.

RESULTS

13a) For each group, the numbers of participants who were randomly assigned, received intended treatment, and were analysed for the primary outcome
NPT: The number of care providers or centers performing the intervention in each group and the number of patients treated by each care provider in each center

Does your paper address CONSORT subitem 13a? *

Copy and paste relevant sections from the manuscript (include quotes in quotation marks "like this" to indicate direct quotes from your manuscript), or elaborate on this item by providing additional information not in the ms, or briefly explain why the item is not applicable/relevant for your study

"The 202 of 216 participants (Figure 1) were recruited over 10 months."

13b) For each group, losses and exclusions after randomisation, together with reasons

Does your paper address CONSORT subitem 13b? (NOTE: Preferably, this is shown in a CONSORT flow diagram) *

Copy and paste relevant sections from the manuscript (include quotes in quotation marks "like this" to indicate direct quotes from your manuscript), or elaborate on this item by providing additional information not in the ms, or briefly explain why the item is not applicable/relevant for your study

The 202 participants ("Figure 1") were recruited over 10 months.

13b-i) Attrition diagram

Strongly recommended: An attrition diagram (e.g., proportion of participants still logging in or using the intervention/comparator in each group plotted over time, similar to a survival curve) or other figures or tables demonstrating usage/dose/engagement.

	1	2	3	4	5	
subitem not at all important	○	○	○	○	○	essential

Does your paper address subitem 13b-i?

Copy and paste relevant sections from the manuscript or cite the figure number if applicable (include quotes in quotation marks "like this" to indicate direct quotes from your manuscript), or elaborate on this item by providing additional information not in the ms, or briefly explain why the item is not applicable/relevant for your study

Attrition was the number of participants who completed the study.

14a) Dates defining the periods of recruitment and follow-up

Does your paper address CONSORT subitem 14a? *

Copy and paste relevant sections from the manuscript (include quotes in quotation marks "like this" to indicate direct quotes from your manuscript), or elaborate on this item by providing additional information not in the ms, or briefly explain why the item is not applicable/relevant for your study

"The study ran from December 2020 to December 2021, during the COVID-19 pandemic"

14a-i) Indicate if critical “secular events” fell into the study period

Indicate if critical “secular events” fell into the study period, e.g., significant changes in Internet resources available or “changes in computer hardware or Internet delivery resources”

	1	2	3	4	5	
subitem not at all important	○	○	○	○	◉	essential

Clear selection

Does your paper address subitem 14a-i?

Copy and paste relevant sections from the manuscript (include quotes in quotation marks "like this" to indicate direct quotes from your manuscript), or elaborate on this item by providing additional information not in the ms, or briefly explain why the item is not applicable/relevant for your study

'The study ran from December 2020 to December 2021, during the COVID-19 pandemic"

14b) Why the trial ended or was stopped (early)

Does your paper address CONSORT subitem 14b? *

Copy and paste relevant sections from the manuscript (include quotes in quotation marks "like this" to indicate direct quotes from your manuscript), or elaborate on this item by providing additional information not in the ms, or briefly explain why the item is not applicable/relevant for your study

This is not applicable to our study.

15) A table showing baseline demographic and clinical characteristics for each group

NPT: When applicable, a description of care providers (case volume, qualification, expertise, etc.) and centers (volume) in each group

Does your paper address CONSORT subitem 15? *

Copy and paste relevant sections from the manuscript (include quotes in quotation marks "like this" to indicate direct quotes from your manuscript), or elaborate on this item by providing additional information not in the ms, or briefly explain why the item is not applicable/relevant for your study

Yes. Table 1: Participant characteristics at baseline

Characteristic Overall
n=202 Arm 1
n=67 Arm 2
n=68 Arm 3
n=67
Age in years, mean (SD) 61·0 (11·1) 61·2 (9·4) 61·4 (11·7) 60·4 (12·1)
Biological Sex, No. (%)
- Female 130 (64·4) 44 (65·7) 47 (69·1) 39 (58·2)
- Male 72 (35·6) 23 (34·3) 21 (30·9) 28 (41·8)

Education, No. (%)
- Some/Completed High School 34 (16·8) 11 (16·4) 14 (20·6) 9 (13·4)
- Some University/College 33 (16·3) 6 (8·9) 14 (20·6) 13 (19·4)
- Completed University/College 95 (47·0) 34 (50·7) 29 (42·6) 32 (47·8)
- Some/Completed Graduate School 40 (19·8) 16 (23·9) 11 (16·2) 11 (16·2)

Annual Income, No. (%)
- < $20 000 9 (4·4) 1 (1·5) 6 (8·8) 2 (3·0)
- Between $20 000 - $39 000 30 (14·8) 11 (16·4) 10 (14·7) 9 (13·4)
- Between $40 000 - $59 000 28 (13·9) 14 (20·9) 8 (11·8) 6 (9·0)
- Between $60 000 - $79 999 26 (12·9) 9 (13·4) 7 (10·3) 10 (14·9)
- Between $80 000 - $99 999 31 (15·3) 9 (13·4) 13 (19·1) 9 (13·4)
- > $100 000 59 (29·2) 21 (31·3) 19 (27·9) 19 (28·3)
- Not Reported 19 (9·4) 2 (3·0) 5 (7·3) 12 (17·9)

..."

15-i) Report demographics associated with digital divide issues

In ehealth trials it is particularly important to report demographics associated with digital divide issues, such as age, education, gender, social-economic status, computer/Internet/ehealth literacy of the participants, if known.

	1	2	3	4	5	
subitem not at all important	◉	○	○	○	○	essential

Clear selection

Does your paper address subitem 15-i? *

Copy and paste relevant sections from the manuscript (include quotes in quotation marks "like this" to indicate direct quotes from your manuscript), or elaborate on this item by providing additional information not in the ms, or briefly explain why the item is not applicable/relevant for your study

This is explored in detail in the protocol paper.

16) For each group, number of participants (denominator) included in each analysis and whether the analysis was by original assigned groups

16-i) Report multiple “denominators” and provide definitions

Report multiple “denominators” and provide definitions: Report N’s (and effect sizes) “across a range of study participation [and use] thresholds” [1], e.g., N exposed, N consented, N used more than x times, N used more than y weeks, N participants “used” the intervention/comparator at specific pre-defined time points of interest (in absolute and relative numbers per group). Always clearly define “use” of the intervention.

	1	2	3	4	5	
subitem not at all important	○	○	◉	○	○	essential

Clear selection

Does your paper address subitem 16-i? *

Copy and paste relevant sections from the manuscript (include quotes in quotation marks "like this" to indicate direct quotes from your manuscript), or elaborate on this item by providing additional information not in the ms, or briefly explain why the item is not applicable/relevant for your study

When reporting, we used multiple denominators where possible. See this example from the Results: "Acceptability: The completion rate for the study was 94·6%, and the mean attendance for Arms 2 and 3 combined was 78·6% for the 5 group nutrition classes, 88·5% for the 12 group exercise sessions, and 78·8% for the home exercise videos, with no statistically significant differences between the two intervention arms. Results from the Satisfaction Survey for the Heal-Me personnel light (Arm 2) and intensive (Arm 3) programs (n=122) were high at 80·6% (SD 19·0) and 85·0% (SD 15·2), respectively (p=0·17). Though staff services were scored as "Excellent" (97/122 or 79·5%), participants in Arm 3 scored staff services statistically significantly higher than participants in Arm 2 (U=2209, z=2·57, p=0·01). At the end-of-study, when participants were asked if Pioneer had prepared them to reach their exercise and protein goals after ..."

16-ii) Primary analysis should be intent-to-treat

Primary analysis should be intent-to-treat, secondary analyses could include comparing only "users", with the appropriate caveats that this is no longer a randomized sample (see 18-i).

	1	2	3	4	5	
subitem not at all important	○	○	○	○	◉	essential

Clear selection

Does your paper address subitem 16-ii?

Copy and paste relevant sections from the manuscript (include quotes in quotation marks "like this" to indicate direct quotes from your manuscript), or elaborate on this item by providing additional information not in the ms, or briefly explain why the item is not applicable/relevant for your study

"The intention-to-treat (ITT) principle was used for all analyses."

17a) For each primary and secondary outcome, results for each group, and the estimated effect size and its precision (such as 95% confidence interval)

Does your paper address CONSORT subitem 17a? *

Copy and paste relevant sections from the manuscript (include quotes in quotation marks "like this" to indicate direct quotes from your manuscript), or elaborate on this item by providing additional information not in the ms, or briefly explain why the item is not applicable/relevant for your study

Yes! See Table 2 which I tried to include here but then ran out of space for the entire application.

17a-i) Presentation of process outcomes such as metrics of use and intensity of use

In addition to primary/secondary (clinical) outcomes, the presentation of process outcomes such as metrics of use and intensity of use (dose, exposure) and their operational definitions is critical. This does not only refer to metrics of attrition (13-b) (often a binary variable), but also to more continuous exposure metrics such as "average session length". These must be accompanied by a technical description how a metric like a "session" is defined (e.g., timeout after idle time) [1] (report under item 6a).

	1	2	3	4	5	
subitem not at all important	○	○	○	○	◉	essential

Clear selection

Does your paper address subitem 17a-i?

Copy and paste relevant sections from the manuscript (include quotes in quotation marks "like this" to indicate direct quotes from your manuscript), or elaborate on this item by providing additional information not in the ms, or briefly explain why the item is not applicable/relevant for your study

"Acceptability: The completion rate for the study was 94·6%, and the mean attendance for Arms 2 and 3 combined was 78·6% for the 5 group nutrition classes, 88·5% for the 12 group exercise sessions, and 78·8% for the home exercise videos, with no statistically significant differences between the two intervention arms. "

17b) For binary outcomes, presentation of both absolute and relative effect sizes is recommended

Does your paper address CONSORT subitem 17b? *

Copy and paste relevant sections from the manuscript (include quotes in quotation marks "like this" to indicate direct quotes from your manuscript), or elaborate on this item by providing additional information not in the ms, or briefly explain why the item is not applicable/relevant for your study

The relative risk and the risk difference were not reported as our results were continuous, categorical, or ordinal.

18) Results of any other analyses performed, including subgroup analyses and adjusted analyses, distinguishing pre-specified from exploratory

Does your paper address CONSORT subitem 18? *

Copy and paste relevant sections from the manuscript (include quotes in quotation marks "like this" to indicate direct quotes from your manuscript), or elaborate on this item by providing additional information not in the ms, or briefly explain why the item is not applicable/relevant for your study

Yes! All exploratory outcomes were separated from the primary and secondary ones under a section header.

"Exploratory outcomes:
One hundred and forty-four participants (71.2%) had 3-day food record completed at both baseline and end-of-study. Analysis of the food records showed that protein and calorie intakes increased in the Heal-Me intervention arms (Arms 2 and 3) compared to controls (Supplementary Table 3)...."

18-i) Subgroup analysis of comparing only users

A subgroup analysis of comparing only users is not uncommon in ehealth trials, but if done, it must be stressed that this is a self-selected sample and no longer an unbiased sample from a randomized trial (see 16-iii).

	1	2	3	4	5	
subitem not at all important	(selected)	○	○	○	○	essential

Clear selection

Does your paper address subitem 18-i?

Copy and paste relevant sections from the manuscript (include quotes in quotation marks "like this" to indicate direct quotes from your manuscript), or elaborate on this item by providing additional information not in the ms, or briefly explain why the item is not applicable/relevant for your study

No, this was not done as the adherence rate to the app exceeded pre-study expectations.

19) All important harms or unintended effects in each group
(for specific guidance see CONSORT for harms)

Does your paper address CONSORT subitem 19? *

Copy and paste relevant sections from the manuscript (include quotes in quotation marks "like this" to indicate direct quotes from your manuscript), or elaborate on this item by providing additional information not in the ms, or briefly explain why the item is not applicable/relevant for your study

"Adverse events: No serious adverse events occurred during the trial. However, 33 minor health/ musculoskeletal complaints (e.g., muscle pain) were reported across participants in the three study groups (control: 10 (30·3%); arm 2: 11(30·3%); arm 3: 12, (39·4%); p=0·708). Twenty-one (63·6%) were reported as ‘clearly unrelated’, five (15·1%) ‘possibly related’, and only five (15·1%) ‘related’ to study participation. "

19-i) Include privacy breaches, technical problems

Include privacy breaches, technical problems. This does not only include physical “harm” to participants, but also incidents such as perceived or real privacy breaches [1], technical problems, and other unexpected/unintended incidents. “Unintended effects” also includes unintended positive effects [2].

	1	2	3	4	5	
subitem not at all important	(●)	○	○	○	○	essential

Clear selection

Does your paper address subitem 19-i?

Copy and paste relevant sections from the manuscript (include quotes in quotation marks "like this" to indicate direct quotes from your manuscript), or elaborate on this item by providing additional information not in the ms, or briefly explain why the item is not applicable/relevant for your study

No privacy breaches or technical problems were encountered during the RCT.

19-ii) Include qualitative feedback from participants or observations from staff/researchers

Include qualitative feedback from participants or observations from staff/researchers, if available, on strengths and shortcomings of the application, especially if they point to unintended/unexpected effects or uses. This includes (if available) reasons for why people did or did not use the application as intended by the developers.

	1	2	3	4	5	
subitem not at all important	◉	○	○	○	○	essential

Clear selection

Does your paper address subitem 19-ii?

Copy and paste relevant sections from the manuscript (include quotes in quotation marks "like this" to indicate direct quotes from your manuscript), or elaborate on this item by providing additional information not in the ms, or briefly explain why the item is not applicable/relevant for your study

These are not included in this RCT but will be part of a companion paper in preparation.

DISCUSSION

22) Interpretation consistent with results, balancing benefits and harms, and considering other relevant evidence

NPT: In addition, take into account the choice of the comparator, lack of or partial blinding, and unequal expertise of care providers or centers in each group

22-i) Restate study questions and summarize the answers suggested by the data, starting with primary outcomes and process outcomes (use)

Restate study questions and summarize the answers suggested by the data, starting with primary outcomes and process outcomes (use).

	1	2	3	4	5	
subitem not at all important	○	○	○	○	◉	essential

Clear selection

Does your paper address subitem 22-i? *

Copy and paste relevant sections from the manuscript (include quotes in quotation marks "like this" to indicate direct quotes from your manuscript), or elaborate on this item by providing additional information not in the ms, or briefly explain why the item is not applicable/relevant for your study

". Acceptability was high across both adherence and satisfaction measures, but the primary effectiveness outcome of self-reported lower extremity function was not significantly different between groups. Statistically significant improvements were found in the Heal-Me intervention for the 2-minute step test, well-being, quality of life, and the proportion of participants reaching target protein intake. "

22-ii) Highlight unanswered new questions, suggest future research

Highlight unanswered new questions, suggest future research.

	1	2	3	4	5	
subitem not at all important	○	○	○	○	◉	essential

Clear selection

Does your paper address subitem 22-ii?

Copy and paste relevant sections from the manuscript (include quotes in quotation marks "like this" to indicate direct quotes from your manuscript), or elaborate on this item by providing additional information not in the ms, or briefly explain why the item is not applicable/relevant for your study

"As well, challenges were noted with participants submitting their 3-day food records, with only 71.2% with complete records both at baseline and end-of-study, suggesting the need to explore additional forms of tracking in future studies29."

20) Trial limitations, addressing sources of potential bias, imprecision, and, if relevant, multiplicity of analyses

20-i) Typical limitations in ehealth trials

Typical limitations in ehealth trials: Participants in ehealth trials are rarely blinded. Ehealth trials often look at a multiplicity of outcomes, increasing risk for a Type I error. Discuss biases due to non-use of the intervention/usability issues, biases through informed consent procedures, unexpected events.

	1	2	3	4	5	
subitem not at all important	○	○	○	○	●	essential

Clear selection

Does your paper address subitem 20-i? *

Copy and paste relevant sections from the manuscript (include quotes in quotation marks "like this" to indicate direct quotes from your manuscript), or elaborate on this item by providing additional information not in the ms, or briefly explain why the item is not applicable/relevant for your study

Limitations
A key limitation of the study was participant selection. Though carried out with sound rationale, we limited enrolment to participants who had graduated from an exercise-based rehabilitation program for their respective chronic diseases. A history of previous exercise participation is associated with self-effectiveness, competence, and adherence to exercise interventions25,26. The step count and moderate-vigorous activity intensity minutes were similar for the intervention and control groups, suggesting..."

21) Generalisability (external validity, applicability) of the trial findings

NPT: External validity of the trial findings according to the intervention, comparators, patients, and care providers or centers involved in the trial

21-i) Generalizability to other populations

Generalizability to other populations: In particular, discuss generalizability to a general Internet population, outside of a RCT setting, and general patient population, including applicability of the study results for other organizations

	1	2	3	4	5	
subitem not at all important	○	○	○	◉	○	essential

Clear selection

Does your paper address subitem 21-i?

Copy and paste relevant sections from the manuscript (include quotes in quotation marks "like this" to indicate direct quotes from your manuscript), or elaborate on this item by providing additional information not in the ms, or briefly explain why the item is not applicable/relevant for your study

Although this was conducted during COVID-19, fully virtual RCTs as well as online health programs are the direction of the future. As such, results can be applied to other chronic disease populations with high levels of smart device ownership and moderate technology proficiency.

21-ii) Discuss if there were elements in the RCT that would be different in a routine application setting

Discuss if there were elements in the RCT that would be different in a routine application setting (e.g., prompts/reminders, more human involvement, training sessions or other co-interventions) and what impact the omission of these elements could have on use, adoption, or outcomes if the intervention is applied outside of a RCT setting.

	1	2	3	4	5	
subitem not at all important	(selected)	○	○	○	○	essential

Clear selection

Does your paper address subitem 21-ii?

Copy and paste relevant sections from the manuscript (include quotes in quotation marks "like this" to indicate direct quotes from your manuscript), or elaborate on this item by providing additional information not in the ms, or briefly explain why the item is not applicable/relevant for your study

None, there would still need to be an orientation, exercise check in, and a nutrition check in at baseline to onboard the participant and establish parameters for their health programs.

OTHER INFORMATION

23) Registration number and name of trial registry

Does your paper address CONSORT subitem 23? *

Copy and paste relevant sections from the manuscript (include quotes in quotation marks "like this" to indicate direct quotes from your manuscript), or elaborate on this item by providing additional information not in the ms, or briefly explain why the item is not applicable/relevant for your study

..."was registered with clinicaltrials.gov (NCT04666558)."

24) Where the full trial protocol can be accessed, if available

Does your paper address CONSORT subitem 24? *

Cite a Multimedia Appendix, other reference, or copy and paste relevant sections from the manuscript (include quotes in quotation marks "like this" to indicate direct quotes from your manuscript), or elaborate on this item by providing additional information not in the ms, or briefly explain why the item is not applicable/relevant for your study

https://pubmed.ncbi.nlm.nih.gov/35569753/

25) Sources of funding and other support (such as supply of drugs), role of funders

Does your paper address CONSORT subitem 25? *

Copy and paste relevant sections from the manuscript (include quotes in quotation marks "like this" to indicate direct quotes from your manuscript), or elaborate on this item by providing additional information not in the ms, or briefly explain why the item is not applicable/relevant for your study

"Funding for this study was provided by a COVID-19 grant from Alberta Innovates Health Solutions."

X27) Conflicts of Interest (not a CONSORT item)

X27-i) State the relation of the study team towards the system being evaluated

In addition to the usual declaration of interests (financial or otherwise), also state the relation of the study team towards the system being evaluated, i.e., state if the authors/evaluators are distinct from or identical with the developers/sponsors of the intervention.

	1	2	3	4	5	
subitem not at all important	◉	○	○	○	○	essential

Clear selection

Does your paper address subitem X27-i?

Copy and paste relevant sections from the manuscript (include quotes in quotation marks "like this" to indicate direct quotes from your manuscript), or elaborate on this item by providing additional information not in the ms, or briefly explain why the item is not applicable/relevant for your study

Some of the authors were developers of the intervention. However, there were not commercial sponsors involved. This is a research tool for use in research.

About the CONSORT EHEALTH checklist

As a result of using this checklist, did you make changes in your manuscript? *

○ yes, major changes

○ yes, minor changes

◉ no

What were the most important changes you made as a result of using this checklist?

This question is not applicable to the submission since no changes were made.

How much time did you spend on going through the checklist INCLUDING making changes in your manuscript *

It took approximately 2 hours to go through this checklist.

As a result of using this checklist, do you think your manuscript has improved? *

- ( ) yes
- ( ) no
- (•) Other: I think it has remained the same.

Would you like to become involved in the CONSORT EHEALTH group?

This would involve for example becoming involved in participating in a workshop and writing an "Explanation and Elaboration" document

- (•) yes
- ( ) no
- ( ) Other:

Clear selection

Any other comments or questions on CONSORT EHEALTH

I think the comments on expected usage (time) and training/onboarding of the eHealth intervention are mandatory as they are all too frequently overlooked in manuscripts.

STOP - Save this form as PDF before you click submit

To generate a record that you filled in this form, we recommend to generate a PDF of this page (on a Mac, simply select "print" and then select "print as PDF") before you submit it.

When you submit your (revised) paper to JMIR, please upload the PDF as supplementary file.

Don't worry if some text in the textboxes is cut off, as we still have the complete information in our database. Thank you!

Final step: Click submit !

Click submit so we have your answers in our database!

Submit Clear form

Never submit passwords through Google Forms.

This form was created outside of your domain. Report Abuse - Terms of Service - Privacy Policy

Google Forms
